# Barriers and facilitators to the successful development, implementation and evaluation of care bundles in acute care in hospital: a scoping review

**DOI:** 10.1186/s13012-019-0894-2

**Published:** 2019-05-06

**Authors:** D. Gilhooly, S. A. Green, C. McCann, N. Black, S. R. Moonesinghe

**Affiliations:** 1 UCLH NIHR Surgical Outcomes Research Centre, Department of Anaesthesia and Perioperative Medicine, University College Hospital, London, NW1 2BU UK; 2 NIHR CLAHRC Northwest London, Imperial College London Chelsea and Westminster Hospital, London, SW10 9NH UK; 3 Department of Health Services Research Policy, London School of Hygiene and Tropical Medicine, 15-17 Tavistock Place, London, WC1H 9SH UK; 4 Division of Surgery and Interventional Science Charles Bell House, University College London, London, W1W 7TS UK; 5 Health Services Research Centre, National Institute for Academic Anaesthesia, Royal College of Anaesthetists, Churchill House, 35 Red Lion Square, London, WC1R 4SG UK

**Keywords:** Care bundles, Evidence-based care, Implementation, Quality improvement, Improvement science, Healthcare improvement, Evaluation, Intervention design

## Abstract

**Background:**

Care bundles are small sets of evidence-based recommendations, designed to support the implementation of evidence-based best clinical practice. However, there is variation in the design and implementation of care bundles, which may impact on the fidelity of delivery and subsequently their clinical effectiveness.

**Methods:**

A scoping review was carried out using the Arksey and O’Malley framework to identify the literature reporting on the design, implementation and evaluation of care bundles. The Embase, CINAHL, Cochrane and Ovid MEDLINE databases were searched for manuscripts published between 2001 and November 2017; hand-searching of references and citations was also undertaken. Data were initially assessed using a quality assessment tool, the Downs and Black checklist, prior to further analysis and narrative synthesis. Implementation strategies were classified using the Expert Recommendations for Implementing Change (ERIC) criteria.

**Results:**

Twenty-eight thousand six hundred ninety-two publications were screened and 348 articles retrieved in full text. Ninety-nine peer-reviewed quantitative publications were included for data extraction. These consisted of one randomised crossover trial, one randomised cluster trial, one case-control study, 20 prospective cohort studies and 76 non-parallel cohort studies. Twenty-three percent of studies were classified as poor based on Downs and Black checklist, and reporting of implementation strategies lacked structure.

Negative associations were found between the number of elements in a bundle and compliance (Spearman’s rho = − 0.47, non-parallel cohort and − 0.65, prospective cohort studies), and between the complexity of elements and compliance (*p* < 0.001, chi-squared = 23.05). Implementation strategies associated with improved compliance included evaluative and iterative approaches, development of stakeholder relationships and education and training strategies.

**Conclusion:**

Care bundles with a small number of simple elements have better compliance rates. Standardised reporting of implementation strategies may help to implement care bundles into clinical practice with high fidelity.

**Trial Registration:**

This review was registered on the PROSPERO database: CRD 42015029963 in December 2015.

**Electronic supplementary material:**

The online version of this article (10.1186/s13012-019-0894-2) contains supplementary material, which is available to authorized users.

## Key points

Question: Which factors affect the implementation of care bundles in clinical practice?

Findings: This scoping review looked both at the compliance of implementation of care bundles and their constituent elements. Ninety-nine quantitative papers were included, which implemented 106 care bundles. We have identified several broad strategies for development and implementation of care bundles which are associated with improved compliance in practice. Care bundles with a few simple elements and using formative evaluations are associated with better compliance in the general acute care setting.

Meaning: These findings should be used to guide the design and implementation of future care bundles.

## Background

Evidence-based medicine can be defined as a systematic approach to clinical problem solving which allows the integration of best available research evidence with clinical expertise and patient values [1]. However, the effective implementation of evidence in clinical practice still presents many challenges to healthcare professionals, illustrated by the vast number of guidelines that are published each year [2]. One mechanism that can support the distillation of this evidence into a more usable and practical form is through the development and implementation of ‘care bundles’.

The Institute for Healthcare Improvement (IHI) has developed criteria required for an element to be included in a care bundle. These are [3]:

1. Robust evidence for the clinical change
2. Little or no controversy concerning their efficacy
3. Consensus and high degree of acceptance

A bundle was defined as ‘A small set of evidence-based interventions for a defined patient segment/population and care setting that, when implemented together, will result in significantly better outcomes than when implemented individually’. The ventilator and central line bundles were the first two to be introduced. The guidelines for the design of each bundle were comprehensive, stating that bundles should only contain three to five interventions with the strong clinical agreement so that implementation would not lead to time lost debating over their validity.

Multiple systematic reviews and meta-analyses have been published investigating both adherence to, and effectiveness of, care bundles addressing a single clinical issue such as sepsis or surgical site infections [4–10]. Only two of these reviews have looked at the implementation of care bundles in the critical care setting and their effect on patient outcomes [11, 12].

Despite the widespread interest in care bundles, it has been stated they are a non-evidenced-based method of prioritising evidence-based recommendations, and therefore, a need remains for a comprehensive review of the development, implementation and subsequent evaluation of care bundles, particularly outside the critical care setting [7]. Furthermore, in the implementation of care bundles, it is important to assess the fidelity of the intervention in order to evaluate the potential reasons for success or failure of both implementation and of clinical effectiveness. This review is the first to specifically look at all care bundles implemented in the acute care setting and to attempt to delineate which factors affect their successful adoption. Given the heterogeneity of published studies as regards both methods and clinical area, a scoping review (rather than systematic review) was the preferred choice [13]. The aim was to summarise a large body of existing literature, through assessment of studies that describe the design, implementation and evaluation of care bundles in a variety of settings to address the following research questions.

1. How are care bundles designed, what evidence is used to select for elements of the care bundle and what strategies are used for development?
2. What strategies/methods are used to support the implementation of care bundles?
3. What factors in design, implementation and evaluation of care bundles influence successful adoption?

## Methods

A scoping review was chosen as this method addresses our aim to provide an overview of a large and diverse body of literature pertaining to a broad topic [14]. Both quantitative and qualitative papers were included in the search strategy, but this paper will only report on quantitative studies. The qualitative studies will be reported elsewhere.

### Study design

A scoping review was conducted to determine facilitators and barriers to the successful development, implementation and evaluation of care bundles in acute care in hospital.

This review was registered on the PROSPERO database under the registration number CRD 42015029963 in December 2015 [15] and conducted according to the standards and guidance set out in the Arksey and O’Malley framework [16].

### Searches

The search was carried out on Embase, Cochrane DARE library, CINAHL and Ovid MEDLINE for all articles relating to the implementation of a care bundle in the acute care setting. As we were not limited to a particular subset of care bundles and in order to try and capture all available evidence, the search was broadened to bundle*, care bundles and patient care bundles. We limited the search to human studies published since the first guidelines by the IHI on care bundles (1 January 2001–1 November 2017). English and European languages were included. Studies were screened for relevance initially by title and abstract; those manuscripts long-listed after this process were then reviewed in full for eligibility. Citations and references of shortlisted publications were searched for further relevant articles using ISI Web of Science.

### Study inclusion and exclusion criteria

We sought to identify published quantitative studies (any study that generated numerical data on the fidelity of care bundle use) that explicitly referred to a care bundle and included a definition of the elements of the bundle, a description of how the care bundle was implemented (a method or technique designed to enhance adoption of a “clinical” intervention, this does not involve ensuring fidelity [17]; implementation was assessed according to the reporting of any methods or strategies or study design that supported the introduction of the care bundle within a clinical or experimental setting) and an assessment of the level of compliance of the care bundle or individual elements of that care bundle (i.e. proportion of patients in whom is was implemented). For a care bundle to be included, it had to consist of at least three elements. The most commonly reported care bundle, sepsis bundle, contains seven elements. Each element should be relatively independent, actionable to all eligible patients and used for a defined population. Any information that was reported about the process of designing the bundle, or the use of an existing bundle that was identified from the literature was documented under care bundle design. Where the level of compliance was referred to but not stated or the elements of a bundle were not clearly described authors were contacted for further clarification.

Papers describing care bundles implemented outside acute hospitals were excluded, as were articles about bundles delivered solely in intensive care units or which involved obstetric, palliative or neonatal care. These areas were not included either because they have already been covered in previous reviews, or because the clinical setting usually has substantially higher or lower staff to patient ratios than routine inpatient care settings, thus limiting the generalisability of findings. Conference abstracts, letters and editorials were also excluded.

### Data extraction

Data were extracted independently from the full-text articles by two of three researchers (DG, SG and CMcC) using a pre-defined extraction sheet. The following data were extracted: author, year of publication, aim, country of study, study design, clinical speciality, care bundle content and design, implementation strategies, compliance, outcome measures, outcome data, limitations and suggested barriers. Discrepancies including missing data or differences in values/interpretation were discussed between DG, SG and CMcC; where agreement could not be reached, SRM was asked to arbitrate.

### Study quality assessment

Although quality assessment is not part of the original scoping review described by Arksey and O’Malley, more recent literature is asserting the necessity of their inclusion using validated tools [18]. Therefore, study quality was assessed using the Downs and Black checklist [19]. In common with many other reviews, we calculated a total score (0–28), which was used to give an indication of the comparative validity and reliability of each study. Studies scoring 19 or more were considered good whereas those less than 14 were considered poor [20]. DG, SG and CMcC extracted data from all identified studies separately and applied the quality assessment. The disagreement was resolved through discussion, and clarification regarding the quality assessment was sought from NB.

### Implementation strategies

Each study was assessed to identify which implementation strategies were used. Strategies were categorised according to the Expert Recommendations for Implementing Change (ERIC) [21], (Additional file 1: Appendix 1A). Where a study used more than one strategy in a particular category, the category was listed once, i.e. if a paper listed different forms of clinician reminders such as posters, internet alerts and case note reminders, this was only listed once for that paper.

### Complexity of elements

The Medical Research Council has published guidance on the evaluation of complex interventions in the health service which was updated in 2008 [22]. The list of what makes an intervention complex be included is shown in Additional file 1: Appendix 1B. Although there is no clear boundary in what separates a simple from a complex intervention, we used these characteristics to evaluate the individual elements of the care bundles included in this scoping review. While some may consider the implementation of a care bundle a complex procedure in itself as it consists of multiple elements, we felt it important to also look at the individual elements themselves to assess their influence on implementation [23]. Therefore, we have used a binary classification (complex vs. simple) to define this, based on the MRC description.

### Data synthesis and presentation

Data analysis was divided into two sections. We have reported narratively because we chose to include both studies that reported compliance for all or part of the individual elements as well as those which reported total care bundle compliance; further, there was substantial heterogeneity of clinical topics, strategies and study designs. For care bundles with total compliance measured, we report the compliance measured at the end of the intervention period, known as resultant compliance. We also report if pre-intervention or baseline compliance was measured to assess improvement in compliance. We have evaluated the effect of implementation strategies on compliance through linear regression. Studies were then stratified by study design and the relationships between compliance and the number of elements in a care bundle assessed using Spearman’s rank order test. For all inferential statistics, in addition to analysing good and fair included studies together, we also conducted sensitivity analyses to establish if there were differences in findings between good, fair and poor quality studies (as evaluated using the Downs and Black criteria).

Analyses were conducted using RStudio version 1.0.136, Boston, USA.

## Results

**Fig. 1:**
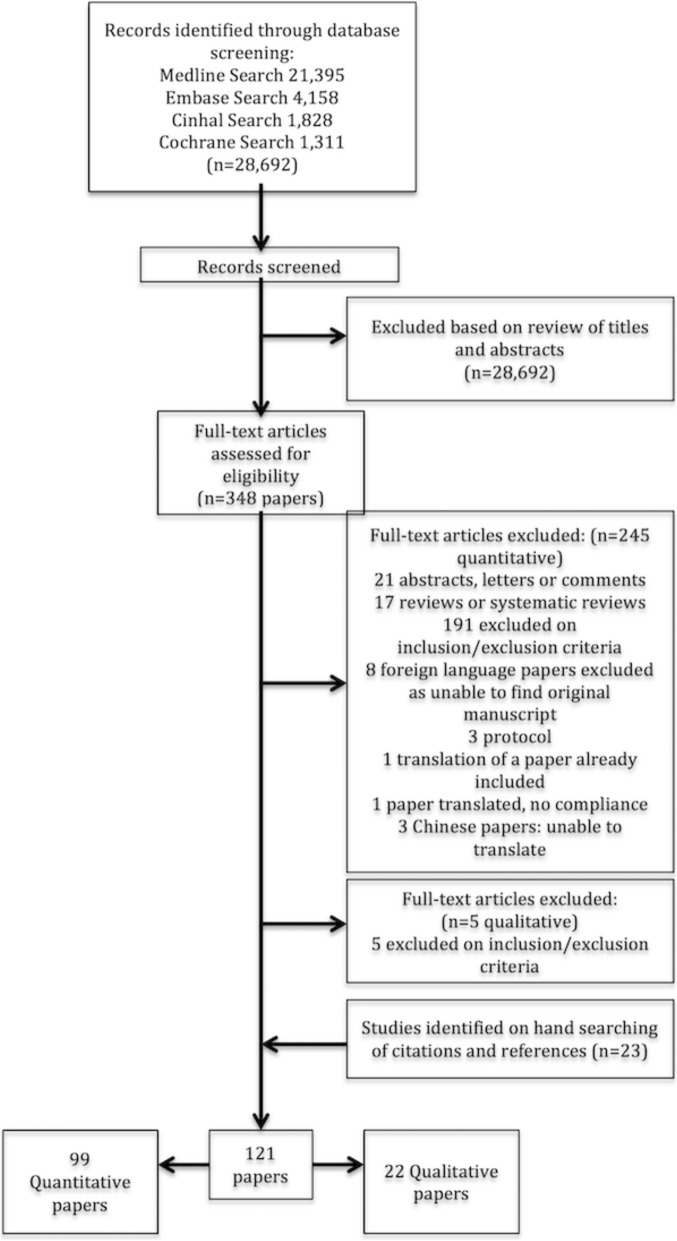
Flow diagram of scoping review

We identified 28,692 articles from our initial searches. After screening titles and abstracts, we identified 348 articles of relevance, which were subsequently retrieved for full-text data extraction (Fig. 1—flow diagram). Ninety-nine papers reporting quantitative studies were included in the final analysis.

### Studies characteristics

Articles reported one randomised crossover trial, 1 randomised cluster trial, 1 case-control study, 4 retrospective cohort studies, 16 prospective cohort studies and 76 non-parallel cohort studies.

Thirty-four studies reported the implementation of the “Surviving Sepsis” resuscitation bundle, 20 studies reported care bundles to reduce surgical site infections and 12 reported care bundles to reduce central line infections. The other 33 studies included a variety of care bundles (Additional file 2: Appendix 2A, table of included papers).

The specialities in which care bundles were most commonly studied were emergency medicine (23 studies), general medicine [21] infection control/microbiology [12] and colorectal surgery [9].

All but two studies were conducted in a single country, one was part of a worldwide study and another focused on five Asian countries (China, India, South Korea, Singapore and Taiwan). Eighteen countries were reported studies and included Australia, Brazil, Canada, China, Denmark, Germany, India, Ireland, Italy, Japan, Netherlands, S. Korea, Singapore, Thailand, UK and USA. The USA was the most commonly represented country (48 studies).

### Quality of studies

Nineteen studies were classified as good, 57 as fair and 23 as poor. Details of each paper’s score, care bundle type, year of publication and country are shown in Additional file 2: Appendix 2B.

### Care bundle design

Care bundle design was addressed in 99 articles. These were divided into 42 that were adopted (used a previously developed care bundle), 23 adapted (used a previously developed care bundle which was altered by the implementation team or developed a care bundle from recognised guidelines) and 34 created de novo (using a combination of guidelines, prior studies, literature reviews) (Table 1).

**Table 1:** List of care bundles based on design

Adopted	42	Resuscitation	28
		Surgical site infection	4
		Central line	3
		Central line bundle for children	2
		Early goal-directed therapy	2
		Falls prevention	2
		Ventilator-associated	1
Adapted	23	Surgical site infection	10
		Central line	4
		Resuscitation	4
		Candidaemia	1
		Falls prevention	1
		MRSA prevention	1
		Peripheral venous cannula	1
		Urinary catheter	1
De novo	34	Surgical site infection	6
		Central line	4
		Acute kidney injury bundle	3
		Antibiotic stewardship	3
		Staphylococcus aureus bacteraemia	3
		Chronic obstructive pulmonary disease	2
		Discharge	2
		Stroke	2
		Bacterial meningitis	1
		Candidaemia	1
		Clostridium difficile	1
		Emergency laparotomy	1
		Hip fracture	1
		MRSA	1
		Peripheral venous cannula	1
		Pharmacy error	1
		Tracheostomy care	1

### Source of content

From a breakdown of care bundles that were created de novo, four studies gave no justification for the care bundle design. Of the remaining 30 studies, a literature review was used in 14, published guidelines or best practice was used in 13 and prior research by the implementation team was used in 6. Other methods included expert opinion, feedback from staff survey, consensus agreement and clinical advisory group. (Details of care bundle development can be seen in Additional file 2 and Additional file 2: Appendix 2A, table of included papers.)

### Number of elements

**Fig. 2:**
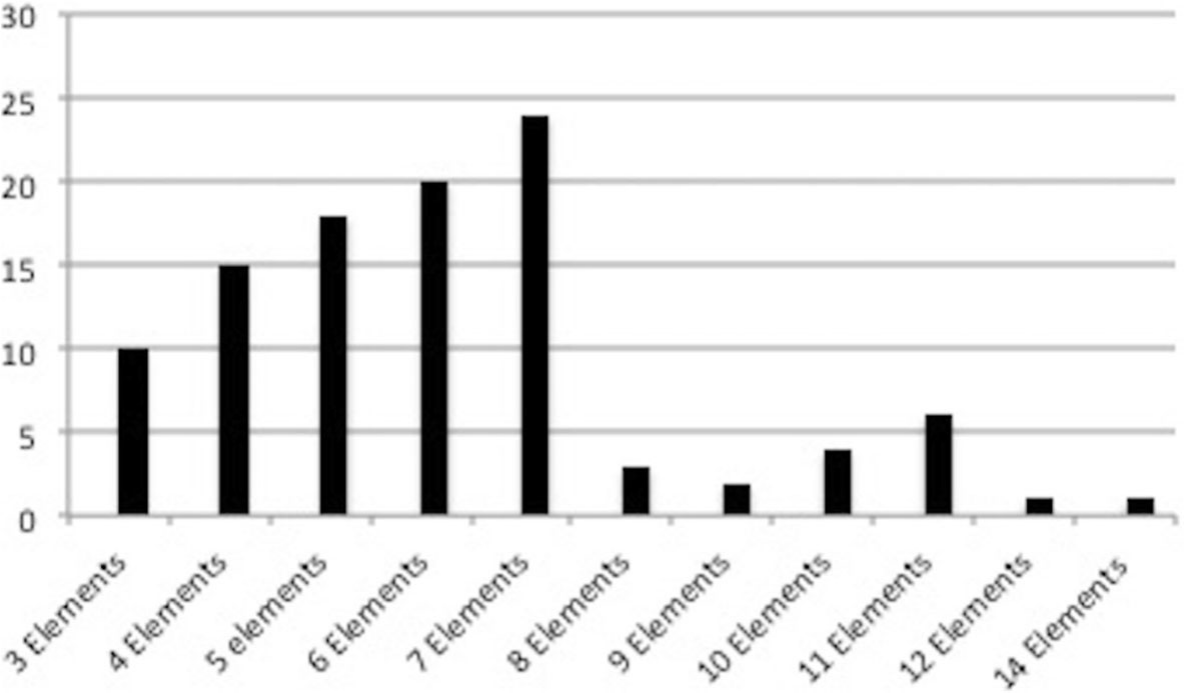
Frequency of element number in the included care bundles. *x*-axis—number of elements in a bundle. *y*-axis—frequency

Two studies reported a variable number of care bundle elements implemented in multicentre studies (Nguyen et al. 2011, 7/8 elements and Bundy et al. 2014, 3–10 elements) and are not included in Fig. 2. Five studies implemented two bundles: Wheeler et al. (insertion and maintenance care bundle for CVC), Takesue et al. (immediate and late care bundle for candidaemia treatment), Sutton et al. (falls prevention care bundle for general and at-risk patients), Bessesen et al. (MRSA prevention care bundle in 2 different hospitals) and Power et al. (early and rehabilitation care bundle for stroke patients). One study, Richardson et al., instituted three falls care bundles (a general care bundle, a care bundle for vulnerable patients and a post fall care bundle).

### Compliance

Forty-six papers listed compliance for some or all of the individual care bundle elements only. Fifty-three studies documented total compliance for the whole care bundle, of which 32 studies had total care bundle compliance as well as documenting compliance for some if not all of the individual care bundle elements. Of those that had full compliance measured, 45 had either a baseline or pre-intervention compliance and a resultant compliance. (Details of compliance can be seen in Additional file 2: Appendix 2A, table of included papers.)

### Complexity of individual elements and compliance

**Fig. 3:**
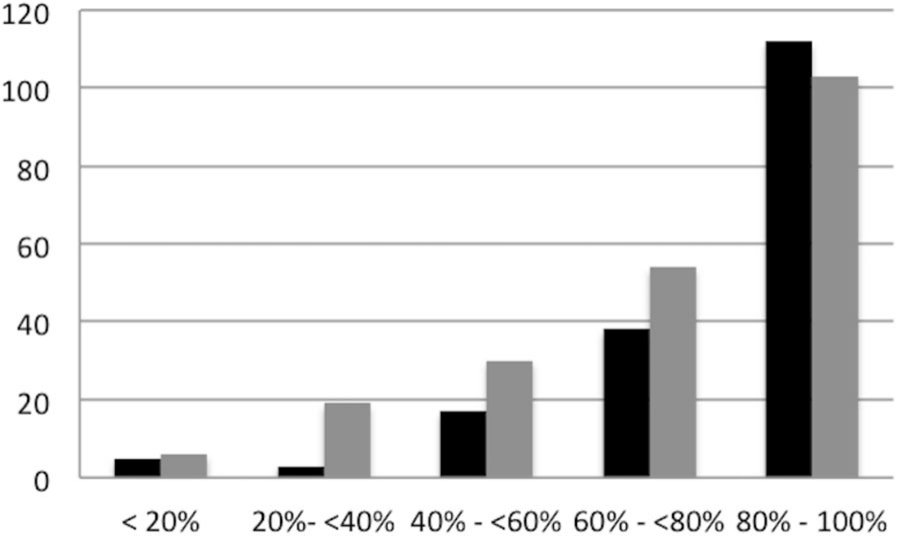
Frequency of element complexity per compliance range. *x*-axis—compliance range. *y*-axis—frequency. Simple. Complex

All the elements with compliance measured individually were listed (*n* = 387). Separating the elements into five equal groups based on compliance, the frequency of complex or simple is shown in Fig. 3.

A significant association was found between worsening compliance and increasing complexity of an element (chi = 23.51, *p* < 0.001) (Kruskal-Wallis).

### Implementation strategies

Forty-eight of the 73 ERIC strategies (Table 2) were used. The most frequently employed strategies were the use of advisory boards (51% of studies, including multidisciplinary teams (MDTs), steering committees, collaborative groups), on-going training (45%), educational meetings (43%) and use of audit and feedback (42%). The number of ERIC strategies used by each study varied between 1 and 13 with a median of 5 strategies.

**Table 2:** Frequency of strategies of implementation categorised according to ERIC classification

Taxonomy of interventions					
Use evaluative and iterative strategies	Audit and provide feedback	42	Develop stakeholder interrelationships	Use advisory boards and workgroups	51
	Develop and implement tools for quality monitoring	25		Identify and prepare champions	26
	Conduct cyclical small tests of change	25		Organise clinical implementation team meetings	8
	Stage implementation scale up	14		Recruit, designate and train for leadership	8
	Purposefully re-examine the implementation	10		Involve executive boards	7
	Develop formal implementation blueprint	10		Obtain formal commitments	6
	Develop and organise quality monitoring systems	9		Build a coalition	5
	Conduct local needs assessment	8		Model and stimulate change	3
	Assess for readiness and identify barriers and facilitators	4		Other	2
	Obtain and use family and patient/consumers feedback	4		Use implementation advisor	1
	Centralise technical assistance	1			
	Capture and share local knowledge	1			
Train and educate stakeholders	Conduct on-going training	45	Provide interactive assistance	Facilitation	15
	Conduct educational meetings	43		Provide clinical supervision	10
	Develop educational material	21		Centralised technical assistance	4
	Make training dynamic	3		Provide local technical assistance	1
	Conduct educational outreach visits	2	Adapt and tailor content	Tailor strategies	14
	Create a learning collaborative	2		Promote adaptability	12
	Use train the trainer strategies	2		Use data experts	1
	Provide on-going consultation	1	Change infrastructure	Change physical structure and equipment	16
	Other	1		Change record systems	3
Support clinicians	Remind clinicians	33		Mandate change	3
	Revise professional roles	23	Utilise financial strategies	Alter incentive/allowance structures	3
	Facilitate relay of clinical data to providers	9		Access new funding	2
	Create new clinical teams	8	Engage consumers	Involve patient and family members	4
	Other	2		Intervene with patients to enhance uptake and adherence	2
				Prepare patients/consumers to be active participants	1

Of the 99 studies, 25 used cyclical small tests of change to improve implementation and another 42 articles stated other formative quality improvement strategies were used such as feedback, addressing barriers, piloting and root cause analysis. This left 32 articles that did not describe any implementation strategies designed to improve care bundle adoption but rather stated conventional implementation strategies such as teaching, education and displaying algorithms. (Details of implementation strategies can be seen in Additional file 2: Appendix 2A, table of included papers.)

### Implementation strategy and care bundle compliance

Fifty-three articles reported care bundle compliance; we have categorised these into high compliance (70–100%), medium compliance (40–69%) and low compliance (0–39%) groups. We excluded three studies from this categorisation: Hauser et al. [24] reported resultant compliance as a range, and Wheeler et al. and Power et al. [25, 26] each implemented two care bundles that fell into different categories. Comparison of compliance against the four main ERIC categories showed higher use of evaluative and iterative strategies (80–95% of high and medium compliance studies versus 76% of low compliance studies), and more development of stakeholder relationships in the high and medium compliance groups (73–76% of high and medium compliance studies versus 48% of low compliance studies), while education and training remains was commonly used in all groups (80–88% of studies across all threes compliance categories). The breakdown of strategies further highlights the high use of champions, MDTs and analysis of results (PDSA cycles within conduct cyclical small test of change and root cause analysis within audit and provide feedback) in high and medium compliance groups compared to low compliance which has higher use of clinician reminders such as posters, printed algorithms and screen savers.

Using linear regression, we assessed the effect of each of the five strategies (champions, MDTs, PDSA cycles, RCA and reminders) on compliance. Univariate analyses found PDSA cycles and RCA to be positively correlated with compliance (correlation coefficient (CC) 0.2 and 0.29 respectively) while reminders were negatively correlated (CC − 0.16). When all these variables were included in a multivariable analysis, only reminders remained weakly negatively correlated (CC − 0.18, *p* < 0.02).

The number of elements in a care bundle was also assessed, and non-parallel cohort and prospective cohort studies were included for sub-analysis as they made up 60% and 32% of the studies, respectively. Three studies were excluded: Hauser et al. [24] as the resultant compliance was reported as a range; Nguyen et al. [27] and Bundy et al. [28] as they implemented a care bundle with a variable number of elements used in each institution. We assessed the number of elements and compliance in good and fair care bundle studies and found a significant inverse association (i.e. the fewer the number of elements, the better the compliance) for both non-parallel cohort and prospective cohort studies, (rho = − 0.47, *p* < 0.02 and rho = − 0.652, *p* < 0.03) respectively. We also found an association between a reducing number of elements and improvement in compliance for non-parallel cohort studies, (rho = − 0.619, *p* < 0.002) from baseline or prior audit, but not for cohort studies, (rho < 0.2, *p* = 0.7). The full table of statistical results can be seen in Additional file 3: Appendix 3A.

### Sensitivity analysis

Sensitivity analysis was done to compare good and fair to poor quality studies. Analysis of implementation strategy and compliance only found MDT and PDSA cycles significantly associated with compliance (CC 0.35 and 0.39, respectively), but none were significant when adjusted. Of note, although reminders were not significant they remained negatively associated when unadjusted. Element number and compliance were only significant for resultant compliance in prospective cohort studies with large effect size (rho = − 0.975, *p* < 0.005).

(Additional file 3: Appendix 3B lists all papers included for statistical analysis).

## Discussion

This scoping review has six key findings. First, the literature on care bundle implementation and evaluation includes a substantial number of low-quality studies, which means that all findings should be interpreted cautiously. Second, although the most frequently used number of elements in a care bundle was seven, using fewer elements was associated with better compliance. Third, elements categorised as simple according to the MRC definitions were associated with higher compliance. Fourth, care bundles with higher compliance were associated with more frequent use of MDTs, champions and formative evaluations as opposed to clinician reminders. Fifth, 48 different implementation strategies, out of a total of 73 described by the ERIC taxonomy, were used in the studies we identified; however, none of the studies included in this review reported the implementation strategies according to these criteria. Finally, the reporting of the fidelity of implementation of care bundles in this literature is variable—only 53 of the 99 papers reported compliance of the whole bundle.

### Designing care bundles

This study has identified that while the majority of peer-reviewed publications assessed report the adoption or adaptation of pre-existing care bundles (65/99), a substantial number were created de novo *(34/99)*. While this finding may in part be due to publication bias, this nevertheless suggests that, although an array of care bundles already exist designed to address a range of clinical and organisational issues in the delivery of care, researchers and practitioners continue to develop novel solutions, often addressing similar problems. While there is substantial literature on the adoption of innovations and the design of interventions [29], how these apply to care bundles is little explored. What is clear is the policy imperative to support the co-design of interventions with front-line healthcare staff and patients. This has been hailed as the key to supporting a more engaged workforce, ensuring that interventions are more likely to be acceptable to staff and patients if they have been involved in designing them [30]. This creates a necessary challenge: co-designing a new intervention for each new setting, clinical team or group of patients requires significant resource and time. Identifying a core intervention with modifiable components that could be co-designed to meets the needs of local staff, patients and the processes that join them has been offered as a potential solution. We have also shown that there is limited patient involvement in care bundle design and implementation.

### Implementation reporting

Clarity is needed to fully understand the “how” when adopting clinical change. We have found that some studies conflate an implementation strategy with the design of an intervention; this therefore emphasises the need for a carefully specified implementation strategy [31]. For example, two studies listed staff education as an element of their care bundle [32, 33]. Although education can be viewed as both an intervention and a form of implementation, having a taxonomy for implementation may support better clarity in reporting. The idea of developing an activity log to aid the clear documentation of implementation strategies has been suggested [34, 35]. Accurate reporting of implementation can also put a burden on authors as publishers enforce word limitations, and this may have a direct effect on replication [36]. This is why we recommend the use of an accepted taxonomy (such as the ERIC criteria) to support authors to ‘speak the common language of implementation’ [31]. The ERIC project initially reviewed the literature, used experts to come to a consensus on detailed definitions for each strategy and then categorised them through concept mapping to arrive at a taxonomy to be used when implementing change [21, 37]. The use of this taxonomy can allow authors to accurately summarise which of the nine strategy categories in manuscript abstracts and go into further detail of the actual strategies in the methods section. If further elaboration is required, then the use of supplements or appendices would be an appropriate way to package them [31]. The Standards for Reporting Implementation Studies (StaRI) Statement further highlights this need and has developed a 27-item checklist to improve the dissemination, implementation and scaling up of interventions. [38]

We were able to show a positive correlation between compliance and both root cause analysis and repeated small cycles of change, but the lack of association found when adjusting for other factors on multivariable analysis may be a result of poor or insufficient reporting of implementation strategies. We did not evaluate a relationship between the number of strategies used and compliance, as this would assume (probably incorrectly) that all implementation strategies have an equal effect. However, we were able to show a significant association between using fewer elements in a care bundle and resultant compliance. Furthermore, the interaction between care bundle elements may change the impact of each intervention as they are implemented simultaneously. This notion is further supported by the qualitative literature: for example, an ethnographic study of the implementation of the sepsis six found that although each element could be viewed as a simple intervention, together, ‘the six’ presents a complex intervention of interdependent tasks, each of which requires prioritisation and scheduling [23]. Thus, there may be a benefit to limiting the number of elements and therefore the potential interactions between them.

### Assessing compliance

Measurement of compliance is an important aspect of care bundle implementation: [3] it is not sufficient to introduce a change and expect it to be fully adopted into routine practice and to attribute improved outcomes to them [39]. Secular change and other factors external to the care bundle can influence outcomes [40]. This begs the question: which is more important, process or outcome evaluation? We argue in favour of measuring both processes and outcomes and being able to evaluate the causal relationship between the two. In this scoping review, only 53 studies reported care bundle compliance, one of which reported it as a range [24].

### Reproducibility

Finally, it is important to be cognisant of the need to evaluate improvement endeavours in resource-limited settings [41]. Of the 99 included articles in this scoping review, although one paper reported it as a worldwide study, only 11 papers reported studies based in 6 low- and middle-income nations; the highest quality manuscript of these was from Uganda [42]. It is important to evaluate and report the different international contexts in which a care bundle can be successfully adapted, and this requires linguistic harmony when describing implementation [31]. The articles discussed in our review, while superficially describing the medical setting, lacked a focus on the structures needed to implement the care bundles.

## Limitations

Although the search strategy was broad and used four search engines as well as reviewing citations and references, some papers that met the inclusion criteria may have been missed. For practical reasons, screening was only undertaken by one reviewer given the number of papers identified. Citation and reference searches were also carried out on all papers included. Papers in Asian languages were not included, with therefore the potential for geographical bias in our results. The heterogeneity of the care bundles precluded in-dept statistical meta-analysis limiting this review to assessing the concept of care bundles. Although most review studies to date have assessed a particular clinical issue allowing more homogeneity in included studies, the assessment of care bundles as a concept will inevitably be heterogeneous. To create some homogeneity, we restricted the review to the acute care setting; however, care bundles implemented in settings with higher staff to patient ratio (for example, intensive care, neonatology and obstetrics) were excluded, in order to reduce the effect of staffing bias on compliance. The inclusion criteria required that compliance must be stated for papers to be included; while we contacted the authors of those that were missing compliance, no further data were available which enabled additional studies to be included. Studies of the implementation of evidence-based care bundles are observational as a randomised trial is considered unethical. For this reason, a scoping review was deemed more appropriate than a systematic review. The results of the sensitivity analysis were unable to robustly defend the results of the main analysis, but this may be because most studies were deemed to be good or fair. This also justifies only good and fair studies being included in the main analysis. Finally, clarity of compliance was variable, with some studies using random samples with or without a strategy, others stated they included all patients but some papers stated neither. We investigated all papers with compliance, but there is a lack of transparency for the exact figures.

## Conclusion

There is a large literature on care bundle implementation in the acute care setting. Summarising this evidence has enabled us to identify several strategies in the design and implementation of care bundles which predispose to success and failure, and in particular ensuring care bundles have a few simple elements so as to increase the likelihood of fidelity. It would help if the reporting of implementation strategies was standardised so as to support future adoption of care bundles. Our findings may be used as a template for future studies, both in terms of design and also description of methodology and findings. If reporting of implementation improves and compliance is correctly reported, future research may help to further elucidate which implementation strategies are most effective for successful care bundle adoption.

## Additional files

Additional file 1: **Appendix 1A.** ERIC classifications. Appendix 1B. Medical Research Council description of a complex intervention (DOCX 51 kb)

Additional file 2: **Appendix 2A.** Summary of included papers. Appendix 2B. Quality of included papers scoring (DOCX 312 kb)

Additional file 3: **Appendix 3A.** Statistical results table. Appendix 3B. List of studies used for statistical analysis (DOCX 116 kb)

## Abbreviations

CC: Correlation coefficient
CVC: Central venous catheter
ERIC: Expert Recommendations for Implementing Change
IHI: Institute for Healthcare Improvement
ISI: Institute of Scientific Information
MDT: Multidisciplinary team
MRC: Medical Research Council
MRSA: Methicillin-resistant *Staphylococcus aureus*
PDSA: Plan, Do, Study, Act
RCA: Root cause analysis
StaRI: Standards for Reporting Implementation Studies
USA: United States of America
