# Supplementary material for: Barriers and facilitators to the successful development, implementation and evaluation of care bundles in acute care in hospital: a scoping review
Source: Implement Sci. 2019 May 6;14:47. doi: 10.1186/s13012-019-0894-2 (PMC6501296; doi:10.1186/s13012-019-0894-2)
Supplement: Supplementary file 1 — Appendix 1A. ERIC classifications. Appendix 1B. Medical Research Council description of a complex intervention (DOCX 51 kb) [file 13012_2019_894_MOESM1_ESM.docx]

**Appendix 1A: Expert Recommendations for Implementing Change (ERIC) classification**

73 implementation strategies were compiled from a review of health and mental health literature and revised by a panel of experts into 9 categories:

1. Use evaluative and iterative strategies
2. Provide interactive assistance
3. Adapt and tailor to context
4. Develop stakeholder interrelationships (between clinical professions)
5. Train and educate stakeholders
6. Support clinicians
7. Engage consumers
8. Utilize financial strategies
9. Change infrastructure

**Appendix 1B: Medical Research Council description of a complex intervention.**

1. Number of interacting components with the experimental and control interventions
2. Number and difficulty of behaviours required by those delivering or receiving the intervention
3. Number of groups or organizational levels targeted by the intervention
4. Number and variability of the outcomes
5. Degree of flexibility or tailoring of the intervention permitted
