# Supplementary material for: Barriers and facilitators to the successful development, implementation and evaluation of care bundles in acute care in hospital: a scoping review
Source: Implement Sci. 2019 May 6;14:47. doi: 10.1186/s13012-019-0894-2 (PMC6501296; doi:10.1186/s13012-019-0894-2)
Supplement: Supplementary file 2 — Appendix 2A. Summary of included papers. Appendix 2B. Quality of included papers scoring (DOCX 312 kb) [file 13012_2019_894_MOESM2_ESM.docx]

Appendix 2A: Summary of included papers

Central line bundles

| **Author and year**  Case number  Element number  *Study design* | Bundle development | Single or multi-centre | Implementation  strategies | Compliance measured | Outcome measured |
| --- | --- | --- | --- | --- | --- |
| **Apisarnthanarak**  **2010**  115 pre  325 post  5 elements  *Non-parallel cohort study* | Previous research- action plan developed by the hospital to bundle evidence based interventions | Single | Phased in  Feedback  Education  Monitored by intervention team | Element 41.3%-81% | CABSI rate per 1000 catheter days pre and post intervention  14 v 1.4  P<0.05 |
| **Bundy 2014**  Unknown  3-10 elements  *Non-parallel cohort study* | Collaborative faculty- The Children's Hospital Association: amalgamation of CDC recommendations and best practice from previous prevention efforts and expert opinion | Multi | Overall collaborative group  MDT formation  6 monthly 2 day reviews on QI, data review and team stories  Monthly webinars  CL maintenance weekly audits | Bundle 86% | CLASBI yearly rate decrease pre and during intervention  10% V 9% p=0.89 |
| **Choi 2013**  235 pre  221 post  5 elements  *Non-parallel cohort study* | Adaption of IHI bundle- team members developed techniques to improve safety and identify best practice | Single | MDT formation  Education / in-service  Root cause analysis for all infections  Monthly meeting with infection rate and RCA  PDSA cycles  Survey on patient safety culture biannually  Adapted for non ICU setting | Element 89%-99%  Bundle 86% | CLA-BSI incidence per 1000-patient days pre and post intervention  2.92 v 1.61 p<0.004 |
| **Duffy 2015**  Random 35 pre and 45 post  5 elements  *Non-parallel cohort study* | CDC guidelines | Single | Identify stakeholders  MDT formation and monthly meetings  Assess Baseline  Education of patients  Training of staff  Integration into education policies  Physician and nurse champions  Identify goals  Audit and feedback  Root Cause Analysis  PDSA  Posters on poorly compliant elements | Bundle 71.2% | CLA-BSI incidence per six months  8v2 p=0.096 |
| **Dumyati 2014**  Unknown  5 elements  *Non-parallel cohort study* | Created a bundle based on literature review and CLABSI prevention guidelines | Multi | Pre-education survey  Introductory lecture and current rates  On-line education (optional to mandatory)  Monthly progress and intervention meetings  MDT formation  On the clock training on wards  Vascular access team  Weekly audits  Assessment of knowledge at end of intervention | Element 82%->90% | CLA-BSI rate per 1000 line days pre and post intervention (95% CI)  2.6 v 1.3 (0.4-0.59) p=0.0179 |
| **Freixas 2012**  **(cvc and pvc) used CVC data**  baseline: 1191843 patient-days, intervention: 1173672 patient-days  7 elements  *Non-parallel cohort study* | Evidence based proposed by coordinating team | Single | Pocket cards  Posters on ward  Training for local trainers  Self assessment questionnaire prior to training  Feedback to ward on adherence and cases of infection  Designated nurse and doctor per hospital to lead project | Element 74.2%-94.8% | CRBSI per 1000 patient days pre and post intervention (95%CI)  0.14 (0.12-0.16) v 0.1 (0.08-0.11) p=0.004 |
| **Grigonis 2016**  6660 pre  6559 post  (Compliance of random weekly sample @ baseline and at 5 weeks)  7 elements  *Non-parallel cohort study* | Adapted from CDC guidelines | Multi | MDT formed  Checklist to track compliance  Webinar for education  Protocol developed  CVC team of nurses who demonstrate competency  Chief of nursing confirmed competent team in each hospital  Quiz to complete on bundle components  Clinical trails manager did onsite compliance visits | Element partial 98-100% | Reduction in mean CLA-BSI rate per hospital  4.5% (95% CI 1.85-7.15) |
| **Jaggi 2014**  Unknown  5 elements  *Non-parallel cohort study* | Based on IHI bundle | Single | Education  Reassess and intervention at 12 months  Develop a central line team  Dedicated CVC trolley  “Scrub the hub” campaign  Teamwork building and development of communication skills | Element 64%-90% | Mean CLA-BSI rate per 1000 catheter days pre and post intervention  5.2 v 2.8 p=0.036 |
| **Kleidon 2014**  42 pre  50 post  5 elements  *Non-parallel cohort study* | Based on literature and prior studies | Single | Baseline audit and feedback  CVC trolley  Education- mandatory electronic  Champions  Feedback- quarterly  QI study | Bundle 58% | CLA-BSI rate per 1000 catheter days pre and post implementation  9.07 v 1.05 p=0.01 |
| **Rinke 2012**  14,987 patient days  6 elements  *Non-parallel cohort study* | CDC recommendations adopted by the Children’s Hospital Association | Single | MDT formation  Monthly root cause analysis meetings  Protected time for nurse engagement  Semi-annual national learning sessions  Continuous QI- display CLASBI and compliance rates  Monthly webinars  Family involvement  Family feedback  Wallet cards to track CL entries | Element 65%-100%  Bundle 65% | CLA-BSI rate per 1000 catheter days (95%CI) pre and post intervention  2.25 (1.02-4.26) v 1.79 (1.06-2.83)  Incidence Rate Ratio 0.8 p=0.58 |
| **Secola 2012**  41 no intervention  41 intervention  7 elements  *Prospective randomized crossover study* | Based on IHI, Child health corporation of America and NACHRI | Single | Training  Education/in-service  CVC team  Blood draw procedure checklist | Bundle 100% | BSI per 1000 catheter days control versus experimental unit  2.1 v 5.7 p=0.97 |
| **Wheeler 2011**  Unknown  4+4 elements  (insertion and maintenance)  *Non-parallel cohort study* | Based on IHI and literature and adopted for children by the Cincinnati Children’s Hospital Medical Centre | Single | MDT formation  Management backing  QI consultant  2 weekly MDT meetings  PDSA cycles  Champions  Weekly report to champions and monthly feedback meetings  Root cause analysis within 48rs of line infection  Education  Set process and outcome goals  Piloting  Empowering staff  Modelled on Breakthrough Series  Change package (key drivers, documentation and aims) | Bundle 100% insertion and 65% maintenance | CA-BSI rate per 1000 line days baseline to end  3.0 v <1.0 |

Sepsis bundles

| **Author and year**  Case number  Element number  *Study design* | Bundle development | Single or multi-centre | Implementation  strategies | Compliance measured | Outcome measured |
| --- | --- | --- | --- | --- | --- |
| **Aguirre-Tejedo**  **2009**  40 cases  8 elements  *Prospective observational cohort study* | Based on SSC | Single | Defined activation code based on clinical criteria  Training of ED staff  Patient care and location prioritized when code activated | Element  Two elements 98% and 100 % | Hospital stay 13+/-8.9 days  Mortality 17.5% (no p value) |
| **Baldwin 2008**  32 cases  8 elements  *Prospective observational audit* | Based on SSC | Single | Education  Electronic guidelines  Laminated posters | Element 50-100%  Bundle 19% | Compliance 19% (no P value) |
| **Berg 2013**  123 cases  7 elements  *Retrospective cohort study* | Based on SSC | Single | Training of advanced practitioners  Development of response team  Protocol for patient identification  Posters of protocol  Monthly protocol review /adjustment | Element  SRT: 25%-47.2%  Non-SRT: 25.3%-96.6%  Bundle 0.8% | In-hospital mortality if SRT activated v not activated  25% v 43.7% p=0.271 |
| **Bruce 2015**  62 pre  133 post  4 elements  *Non-parallel cohort study* | Based on SSC | Multi | Nurse led initiation of protocol  MDT formation  Education  Assigned nurses (staff allocation)  Posters with algorithm  Regular feedback | Element  77.3%-99% | Mortality 24.4% v 21.3%  P= 0.838  Hospital LOS  (pre and post implementation)  8v8 days (p=0.762) |
| **Casserly 2011**  87 cases  3 elements  *Prospective cohort study* | Based on EGDT by Rivers et al. | Single | Combined ED and ICU education  Training ED staff in CVP and EGDT  Training in enhanced communication  Assistance to ED by ICU initially  Sepsis bleep  Monthly audit/feedback/PDSA | Bundle 50% | Time to intervention (min)  Fluid 24 v 54 min  P=0.02  Catheter insertion 142 v 262 min  P=0.01 |
| **Coba 2011**  498 cases  7 elements  *Prospective cohort study* | Based on SSC | Single | Steering committee  Sepsis co-ordinator  Pharmacy order sets  Algorithm pocket/badge cards  Sepsis phone line to pharmacy  **Institutional continuous quality improvement programme**  **1. assessemnt of hospitals preexisting incidence of sepsis and mortality rate**  **2. refinement of methods of early detection of sepsis,**  **3. education on bundle,**  **4. assessment of compliance, 5. quantification of healthcare resource consumption,**  **6. aseesssment of patient outcomes** | Element 34%-93%  Bundle 24.85% | Absolute PMR  29.5% v 9.1% p<0.01 |
| **Daniels 2011**  567 cases  7 elements  *Prospective observational cohort study* | Based on SSC and NHS sepsis six | Single | Education program with exam  Escalation policy  Development of sepsis team  Electronic flagging system  Regular physician education  Buy-in from all stakeholders | Bundle 31.4% (sustained) | Mortality (receiving the sepsis bundle versus not)  51% V 5.9%  P<0.001 |
| **De Miguel-Yanes 2009**  53 pre  50 post  5 elements  *Non-parallel cohort study* | Based on SSC | Single | Education of new junior physicians (part of QI to improve resus bundle uptake by residents)  Education of staff and proficient course  Electronic guidelines for diagnosis and treatment  Segregated environment for treatment | Element 46%-70%  Bundle 26% | Mortality (pre and post implementation)  22.6% v 18%  P=0.62 |
| **Ferrer 2008**  854 pre  1465 post  6 elements  *Non-parallel cohort study* | Based on SSC | Multi | Educational program and presentation  Area coordinators and PI  Regular meeting with coordination centre  Hospital manager meeting prior to start  MDT formation  PI as local champion  Posters and pocket cards of bundle  Audit and feedback | Element 10%-68.9%  Bundle 10.9% | Mortality (95% CI) (pre and post implementation)  44% (41-47%) v 39.7% (37-42)  P=0.04 |
| **Flynn 2015**  59 pre  49 post  3 elements  *Non-parallel cohort study* | Based on SSC- Developed coordinated response to sepsis | Single | MDT formation  Barrier identification and solving  Development of electronic order set  Pharmacist and nurse responders  Sepsis carts | Element 53.1%-87.7% | Length of stay  (IOR) 15 (8-44) v 12(7-12) p=0.14  Mortality  OR (95% CI) (pre and post implementation)  54.2% v 48.9%  0.5 (0.2-1.2) |
| **Girardis 2009**  67 cases  5 elements  *Prospective observational cohort study* | Based on SSC | Single | Education  Protocol for recognition and management (printed and electronic)  Refresher courses, lectures and practical training  Sepsis team  Sepsis team activity documented and discussed with management | Element 70%-100%  Bundle 60% | Mortality (pre and post implementation)  OR 0.15  95% CI 0.03-0.63  P=0.01 |
| **Jeon 2013**  163 pre  203 post  7 elements  *Non-parallel cohort study* | Based on SSC | Single  All wards | Educational program  Conference lectures  Bedside teaching  Simulation | Individual  (partial)  70.9%-96.6% | Length of stay  (IQR) 14 (9-28) v 12 (8-20) p=0.047  Mortality in-hospital (pre and post implementation)  18.4% v 11.8% p=0.078 |
| **Kakebeeke 2013**  323 cases  6 elements  *Prospective observational cohort study* | Based on SSC | Multi  ED | Presentation  Champion  Motivation of staff to all ED goals  Leaflets with inclusion criteria  Part of QI programme but no QI strategies described | Bundle 24% | Achieving full compliance and organ failure, OR (95%CI)  Resp difficulty 3.38 (1.08-10.64)  Hypotension 2.37 (1.07-5.23)  Altered mental status 4.18 (1.92-9.09) |
| **Kalich 2016**  62 Pre  62 Post  10 elements  *Non-parallel cohort study* | Based on SSC and adapted for specific antibiotic guidance | Single  ED | Education via conference and one on one teaching  Posters, badge cards  Mobile app developed | Element partial 14.5%-81% | Appropriate and timely antibiotic administration  OR 1.71 (95% CI 0.62-4.92 p=0.8) |
| **Kang 2012**  317 cases  7 elements  *Retrospective observational cohort study* | Based on SSC | Single  ED | Education program  Protocol for early recognition  Protocol for appropriate management | Bundle 26.8% | Compliance- factors associated with. OR (95%CI)  Hyperthermia 1.37 (1.1-1.7)  Experienced nurses 1.69 (1.1-2.58)  Senior physicians 3.68 (1.68-6.89)  Cryptic shock 0.26 (.013-.052)  High lactate 0.9 (0.82-0.98) |
| **Kim 2014**  88 pre  87 post  4 elements  *Non-parallel cohort study* | Based on SSC | Single | MDT formation  Education | Element 83%-99%  Bundle 80% | 28 day Mortality  (pre and post implementation)  16% v 32% P=0.04 |
| **Kuan 2013**  117 cases  7 elements  *Prospective observational cohort study* | Based on SSC | Single  ED | Education  Lectures and small group teaching  Feedback- mini lectures and bedside laminates  PDSA for barriers quarterly | Bundle 40% | Mortality-bundle complete versus not  11.1% v 18.2% p=0.15 |
| **Laguna-Perez**  84 pre  41 post  7 elements  *Non-parallel cohort study* | Clinical pathway based adapted from SSC | Single  All wards | Education  Training program  Electronic reference guides and protocols  Posters with protocol algorithm | Element 44%-78%  Bundle 0% | Standardized mortality ratio  (95% CI)  Pre V Post implementation  0.76 (0.68-0.87) v 0.6 (0.47-0.82) |
| **Levy 2010**  15,022 cases  7 elements  *Prospective observational cohort study* | Based on SSC- local protocol development | Multi | Local champions identified and trained  Sample screening tool  Campaign manual  Web based directions for implementation  Supportive data for the bundle  Cards and posters of bundle  Feedback to clinicians  Data collection tool  Designated primary reference for all questions  Initiating meetings- institutional change concepts and examples of implementation  QI with SCC and IHI | Element 13.3%-78.7%  Bundle 31.3% | Unadjusted mortality decrease over 2 years (95% CI)  5.4% (2.5%-8.4%) |
| **Liu 2016**  5942 Pre  6544 Post  3 elements  *Non-parallel cohort study* | Adapted SCC bundle | Multi  ED | Sepsis champions  QI staff and operational leadership education  Regional sepsis meeting  Electronic sepsis scorecard developed  Addressed QI and performance improvement  Monthly performance metrics | Element 63.1%-95.9%  Bundle 44.9% | Hospital mortality rate pre and post 9.3% v 7.9% p=0.02  OR .86 (95% CI 0.77-0.97 p<0.01) |
| **MacRedmond 2010**  37 Pre  37 Post  7 elements  *Non-parallel cohort study* | Based on SCC but examined pathway to improve work flow | Single  ED | MDT  Address barriers  PDSA cycles  Education  Pilot launch  Introduction at grand rounds  Collaborative between ICU & ED developed  Electronic order set  Readily available antibiotics | Element partial  62.2%-100% | Crude hospital mortality (pre- and post intervention) 51.4% v27%  Absolute risk reduction (95% CI)  24% (3%-47%) |
| **Micek 2006**  60 pre  60 post  8 elements  *Non-parallel cohort study* | Based on SSC- orderset developed | Single  ED | Education and training  Practical training on equipment  Informed of process and procedures on order set | Element 20%-88.3% | Length of stay  12.1 +/-9.2 d v 8.9 +/-7.2d p=0.038  In-hospital mortality  48.3% v 35% p=0.139  (pre- and post intervention) |
| **Nguyen 2007**  330 cases  5 elements  *Non-parallel cohort study* | Based on SSC and IHI guidelines and survey to ED doctors for validity and feasibility | Single  ED | Assured local acceptance  Sourcing needed equipment  Education- conference, bedside, in-service  Audit  Bundle toolkit in the ED  Feedback- department and individual  PDSA  Champions  Pocket cards of bundle and treatment  Dedicated data extractor | Element 53.7%-90.2%  Bundle 51.2% | In-hospital mortality with completion of the bundle odds ratio (95%CI)  0.36 (0.17-0.79) |
| **Nguyen 2011**  556 cases  7/8 elements  *Prospective observational cohort study* | Based on SSC | Multi  ED | Education  Champions  Weekly audits of implementation  MDT in 3 of 8 hospitals  QI phases 1-4 but no description | Element 63.3%-100%  Bundle 43.6% | Mortality RR reduction with use of modified versus primary SSC bundle (95%CI)  1.94 (1.45-39.1) |
| **Noritomi 2014**  203 baseline  1917 post  6 elements  *Non-parallel cohort study* | Based on SSC | Multi  All wards | Single centralized administration  MDT formed  Bimonthly MDT meeting  Developed screening tool  Assigned responsibility  Guidelines for implementation  Address barriers  Local champion for data collection, QC and questions  Posters printed of productivity  Case manager oversaw every case  Feedback from central team on compliance, mortality and benchmarking | Element 38%-99% Bundle 62% | Mortality risk ratio with bundle completion (95%CI)  0.74 (0.56-0.94) p=0.02 |
| **Palleschi 2014**  50 baseline  100 post  7 elements  *Non-parallel cohort study* | Based on SSC | Multi  All wards | Electronic alert for identification  Education: inter-professional  Training on sepsis care and treatment  Mandatory self learning modules  Poster and badges on treatment guidelines | Element partial  75.4%-88.6% | Phase 1 versus Phase 2  Lactate 50% v 88.6% p<0.000  Blood cultures 72% v 75.4% p<0.054  Antibiotic time (min) (SD)  182.09 (243) v 92.6 (167.9) |
| **Plambech 2012**  27 baseline  29 @18 weeks  48 @ 1 year  6 elements  *Non-parallel cohort study* | Based on SCC and regionally developed Zealand clinical department guidelines | Single  ED | Education and training program  Electronic guidelines  Posters with diagnostic and treatment guidelines  Pocket reference and checklists  MDT formation  Break through series  PDSA for each element  Feedback via graphs displayed  Nurse and doctor champions | Element 13%-75% Bundle 4% | Compliance with 6 elements baseline to one year 7% versus 4%  Non-significant |
| **Ramsdell 2017**  48 pre  110 post  7 elements  *Non-parallel cohort study* | Based on SSC | Single  ED & ICU | MDT formed  Embed documentation tools in E.H.R  Templates to assist providers document fluid status  EWS implemented  Alerts on EH.R  Checklist for nurses to complete  Education | Element 34.8%-90%  Bundle 51.8% | In-hospital mortality rate pre and post intervention  27.1% v 14.5% p=0.05 |
| **Seoane 2013**  1,105 cases  7 elements  *Prospective observational cohort study* | Based on SSC | Single  ED | MDT team developed  Educational program  Monthly MDT meetings to address barriers and opportunities to improve  Rapid cycle feedback/PDSA cycles  Steering committee  Collaboration between ED and ICU formed  Role assignment  Protocols for bundle elements  Goal setting | Bundle 60% | Mean LOS from baseline to 3 yrs later (range)  8 (1-54) v 7 (1-33) p=0.036 |
| **Shin 2013**  770 cases  7 elements  *Retrospective observational cohort study* | Based on SSC | Single  ED | Education program  Conference lecture, bedside teaching and simulation | Bundle 25.6% | OR of 10% increase in ED occupancy rate and overall compliance (95% CI)  0.90 (0.84-0.96) p=0.002 |
| **Tromp 2010**  159 pre  666 post  6 elements  *Non-parallel cohort study* | Based on SSC, expert opinion and nursing staff | Single  ED | MDT formation  Development of screening tool  Nurse practitioner back up  Nurse allow to order CXR  Electronic protocol  Nurses emailed with screening instruction  Nurse champion  Education  Feedback- lecture, email and poster  Individual feedback | Element 48.9%-86.3%  Bundle 12.4% | In-hospital mortality between period one and three  6.3% v 5.5% P>0.05 |
| **Wang 2013**  78 pre  117 post  7 elements  *Non-parallel cohort study* | Based on SSC | Single  ED | Education- definition, recognition and treatment  Protocol for recognition and treatment  Decision making algorithms  Survey of why physicians didn’t complete the bundle  Posters | Element  Bundle 9% | In-hospital mortality (pre- and post intervention)  44.8% v 31.6% P<0.05 |
| **Westphal 2011**  102 pre  115 post  7 elements  *Non-parallel cohort study* | Based on SSC | Multi  All wards | Addition of active surveillance to bundle  Nurses trained in early identification of sepsis | Bundle 28.7% | In-hospital mortality pre and post intervention  61.7% v 38.2% P<0.001 |
| **Whippy 2011**  Unknown  7 elements  *Non-parallel cohort study* | Based on SSC- implemented independently using a playbook- locally refining treatment algorithm | Multi  ED | Sepsis steering committee, project managers and data analysts  MDT formation  Pilot phase  Develop SOP book  Yearly sepsis summit  Local management of ED to ICU transfer  Education and training  PDSA cycles  Champions  Monthly meetings re-barriers  Equipment purchased  Twice monthly sepsis faculty calls for clinical champions | Element 75.4%-97%  Bundle 64% | Risk adjusted sepsis mortality  Decreased but no results printed |

Surgical site infection bundles

| **Author and year**  Case number  Element number  *Study design* | Bundle development | Single or multi-centre | Implementation  strategies | Compliance measured | Outcome measured |
| --- | --- | --- | --- | --- | --- |
| **Bull 2011**  180 pre  275 post  5 elements  *Non-parallel cohort study* | Based on published literature and consensus views on feasibility | Single | Steering committee  Stakeholders identified  Focus groups to discuss progress  Project officer to oversee compliance | Element 31.6%-86.6%  Bundle 21.1% | Infection rate pre and post intervention (95% CI)  15% (10.4-20.2) v 7% (3.4-12.6) |
| **Crolla 2012**  1537 cases (Compliance measured on 10 patients quarterly from June ‘09-Oct ’11)  4 elements  *Non-parallel cohort study* | Based on Dutch hospital patient safety programme | Single | Management support  Full time infection control nurse  Feedback meetings and PDSA  Elements phased in  News letter  Promoted safety culture  RCA for door openings  MDT meeting | Element 80%-100%  Bundle 80% | Infection rate adjusted OR between baseline and end (95% CI)  0.64 (0.44-0.95) p=0.025 |
| **Fisher 2016**  545 pre  324 post  10 elements  *Non-parallel cohort study* | Bundle adopted from National Collaboration of Children’s Hospitals | Single | Integrated bundle into electronic health record  Reported compliance to collaborative network  Benchmarking compliance  Analysis of monthly data by central committee and fed back to individuals  Areas of persistent non compliance had re-education  RCA for infections | Bundle 77% | Mean infection rate per 100 operations pre and post  1.68% v 0.87% (no p value) |
| **Forbes 2008**  105 pre  103 post  3 elements  *Non-parallel cohort study* | Evidence from literature, data from cohort 1 and expert opinion | Single | MDT formation  Pre-intervention survey and analysis to implement change  Protocol formation  Therapy standardized  Introduction at staff meetings  Nurse champions  Monthly performance figures posted in OR  Independent test of change by working group | Element 85.6%-97.6% | Superficial infection rate pre and post intervention  14,3% v 8.7% p=0.21  RR (95%CI)  0.61 (0.28-1.33) |
| **Gould 2016**  88 pre  126 post  12 elements  *Non-parallel cohort study* | Institution developed based on literature review, guidelines and review of hospital data | Single | PDSA  MDT team  Gap analysis  Mapping processes to ID areas of improvement  Parents of patients interviewed  Education  Education material to carers and teaching Teach-back tool  Specialised nursing unit  RCA  Addressed support for care givers/parents | Element partial 98%-100% | SSI infection rate per 100 surgeries pre and post intervention  8.2 v 2.4 p=0.0695 |
| **Hedrick 2007**  379 baseline  390 post  5 elements  *Non-parallel cohort study* | Based on SCIP  Team chose 5 process measures and all approved by OR team | Single | MDT formation  Team champioins  Feedback with monthly infection rates  Assigning responsibility for each element  Visible reminders on charts for bundle  Adapting environment to make completion of bundle easier  Key changes implemented- protocols for antibiotics, normothermia and glucose control | Element 36%-97% | Infection rate Baseline v end  9.2% v 5.6 % p=0.07 |
| **Larochelle 2011**  706 cases  4 elements  *Non-parallel cohort study* | Based on SCIP | Single | Development of order set  Improved antibiotic access  MDT formation  Audit  Feedback  PDSA cycles | Element 70%-100% | Infection rate baseline versus end – no infection rate figures but result non-significant  P=0.84 |
| **Liau 2010**  1040 pre  2,408 post  5 elements  *Non-parallel cohort study* | Institution identified factors causing SSI and project team voted on which ones to work on | Single | Replacement of razors with clippers  Posters to remind patients not to shave  Guidelines for antibiotics distributed  Pro forma for antibiotic use  Posters with guidelines  Antibiotic stock adapted for guidelines  Patients flagged if to get the bundle  Formation of project team  Workflow redesign to embed new interventions, piloted  PDSA cycles | Element 44%-91% | Infection rate Pre and post intervention  3.1% v 0.5% p<0.001 |
| **Lippitt 2017**  91 pre  128 post  5 elements  *Non-parallel cohort study* | Based on institutions own colorectal bundle | Single | MDT formed  Goal setting  Identified modifiable risk factors  Elements added to surgical posting forms  Literature developed for patients  Training & education  Patient’s informed of side effects of bowel prep and given anti-emetics  Elements integrate into E.H.R. | Element partial 50%-97% | SSI rate pre and post intervention  OR 0.13 (95% CI 0.037-0.53 p<0.001) |
| **Pastor 2010**  491 case  (238 cases 1^st^ 14 months and 253 cases 2^nd^ 14 month period)  7 elements  *Prospective cohort study* | Based on SCIP | Single | MDT formation  Monthly meeting to monitor compliance and address barriers (PDSA)  Automatic reminders for re-dosing  Equipment for warming in holding bay  Education/in-service | Bundle 68% | Infection rate 1^st^ 14 months v 2^nd^ 14 months of implementation  19% v 19% p=0.9 |
| **Perez-Blanco 2015**  218 pre  124 post  6 elements  *Non-parallel cohort study* | Based on SCIP | Single | Protocol presentation at two clinical sessions | Element partial  81.1% | Infection rate  27.5% (21.6-33.4) v 16.9% (10.3-23.5) p=0.03  Mortality pre and post intervention (95%CI)  9.2% (5.4-13) v 3.2% (0.1-6.3) p=0.04 |
| **Ryckman 2009**  Unknown  6 elements  *Non-parallel cohort study* | Adapted from adult SCIP- team developed evidence based paediatric SSI bundle | Single | MDT formation  CEO as champion  Identify failure point  Electronic reminders for antibiotic administration  Identifying patients who will require antibiotics (on OT list and patient bracelets)  Availability of antibiotics for cases  Integrating into surgical time out  Redosing algorithm in anaesthetic charts  Feedback daily with team leaders  Daily feedback of concerns of potential failures with team leaders and process mapping | Element partial  91%-94% | Infection rate pre and post intervention  64% reduction in infection rate |
| **Schiavone 2017**  115 pre  118 post  11 elements  *Non-parallel cohort study* | Developed by institution based on literature review, surgeons preferences and hospital guidelines | Single | MDT formed  Technical expert meetings  Assess baseline data  Agreement with committee members on variations of the bundle  Incorporated into E.H.R  Developed high and low risk bundle  Antibiotic guidelines approved by hospital clinical council  RCA  MDT monitoring and response using phased approach  Goal setting | Element partial 89% | SSI pre and post intervention  37% v 12% p<0.001  Wound dehiscence at 30 days pre and post intervention  26% v 2% p<0.001 |
| **Schriefer 2017**  154 pre  387 post  (Compliance measured on a non specified 7 month period)  11 elements  *Non-parallel cohort study* | Developed by institution based on literature review, review hospital data and meeting with technical experts | Single | MDT team formed  Teams in four groups to focus on different aspects of implementation  Reinforced hand hygiene and audited  Hand sanitizer given to patients | Element partial 92%-100% | SSI pre and post intervention  4% v 0% (no p value) |
| **Tanner 2016**  127 pre  166 post  9 elements  *Non-parallel cohort study* | Based on SCIP and integrated into checklist | Single | Senior staff support  Monthly feedback to staff | Element 35%-100%  Bundle 19% | SSI rates pre and post intervention  24% v 28% p>0.05 |
| **Tillman 2013**  Unknown snapshot  3 elements  *Non-parallel cohort study* | Based on SCIP and integrated into checklist | Single | MDT formation  Integrated into surgical safety checklist  Pilot of the study  Education and training  Monitoring and coaching teams post implementation  Use elements that could be easily verbally verified  Surgical team members survey distributed 1 month before and 1 year after implementation | Element 97.7%-98.7% | Infection rate pre and post intervention  3.13% v 2.96% p=0.72 |
| **Toltzis 2014**  Unknown  3 elements  *Non-parallel cohort study* | Based on SCIP and literature- devised by Ohio Children’s Hospital Solutions for Patient Safety | Multi | MDT formation  Team trained in model for improvement  Group training with monthly conference calls  Bi-annual face-2-face meetings  Feedback  Outcomes monitored by program staff  Root cause analysis of each SSI  QI programme | Element partial  78.7% | Unadjusted OR of centre achieving zero SSI during the intervention period (95%CI) versus baseline  3.86 (2.46-6.11) |
| **Van der Slegt 2013**  720 cases  (10 random procedures observed quarterly)  4 elements  *Non-parallel cohort study* | Based on SCIP and literature- developed by the Dutch hospital patient safety programme | Single | MDT formation  Bundle feedback using newsletter with recommendations  Full time infection control nurse appointed  Time out to include bundle  PDSA cycles  Root cause analysis for poorly compliant elements and recommendations  Personal feedback when bundle adherence at risk | Element 70%-100%  Bundle 60% | Infection rate  Year 1 versus year 3 (95%CI)  14.9% v 8.4%  RR 0.57 (0.31-1.03) p=0.043 |
| **Wick 2008**  298 cases  10 elements  *Non-parallel cohort study* | Based on SCIP, IHI and university guidelines | Single | MDT formation  Infection control person with allocated time  PDSA cycles  Education/ in-service | Element 29%-100% | Infection rate 18% overall |
| **Wick 2012**  278 baseline  324 cases  6 elements  *Non-parallel cohort study* | Feedback from staff survey on areas of concern | Single | MTD formation  Steering committee to review progress and identify concerns  Monthly MDT meetings  Champions and team coaches  Executive support  Education/in-service  Leadership team-introduction to education  PDSA cycles | Element partial  92%-95% | Mean infection rate pre and post intervention (95%CI)  27.3% v 18.2%  RR 0.67 (9-58%) p<0.05 |

Other care bundle:

Acute kidney injury bundle

| **Author and year**  Case number  Element number  *Study design* | Bundle development | Single or multi-centre | Implementation  strategies | Compliance measured | Outcome measured |
| --- | --- | --- | --- | --- | --- |
| **Joslin 2015**  100 pre  92 post  11 elements  *Non-parallel cohort study* | Developed in hospital from NCEPOD report | Single | MDT formation  Piloting with changes made  Posters, screen savers  Electronic guidelines  Feedback of baseline audit results  Paper guidelines  Trainee survey of education | Element 45%-89%  Bundle 8.4% | Recognition of AKI  59% v 75% p<0.001  Mortality pre and intervention  12% v 10% no P value given |
| **Kolhe 2015**  1209 pre  1291 post  6 elements  *Non-parallel cohort study* | Developed in hospital from NCEPOD report | Single | Electronic guidelines  Electronic surveillance and alerting  Incorporating CB into electronic record  Education  Posters of algorithm  Interruptive electronic alerts | Element <12%-86.2%  Bundle 21.6% | In-hospital mortality  18% v 23.1% p=0.046  Progression to higher AKI stage  3.9 v 8.1% p=0.01  Odds of death at 30 days (95%CI)  0.7 (0.527-0.95)  pre and during intervention |
| **Kolhe 2016**  3351 cases with 3717 events  6 elements  *Prospective cohort propensity matched study* | Developed in hospital from NCEPOD report | Single | Interruptive alert and request to complete bundle  4 monthly education of junior doctors | Bundle 25.5% | In-hospital mortality  OR 0.76 (95%CI 0.62-0.92) |

Antibiotic stewardship bundle

| **Author and year**  Case number  Element number  *Study design* | Bundle development | Single or multi-centre | Implementation  strategies | Compliance measured | Outcome measured |
| --- | --- | --- | --- | --- | --- |
| **Brumley 2016**  83 baseline  89 post  6 elements  *Non-parallel cohort study* | Developed from institutions guidelines | Single | AST formed  Pharmacy surveillance of patients  Order-set developed | Element partial 53%-100% | Compliance for 3 elements of the bundle pre and post  45% v 81% (p<0.001) |
| **Carreno 2016**  162 pre  124 post  4 elements  *Non-parallel cohort study* | Developed in hospital | Single | 3 pharmacists recruited for self directed learning  Education and training  Audit  Feedback  Electronic clinical decision support  Pharmacists on rounds | Element 81.6%-98.4%  Bundle 68.5% | In-hospital mortality pre and post intervention  4.8% v 7.4% p=0.375 |
| **Toth 2010**  80 pre  80 post  4 elements  *Non-parallel cohort study* | Based on literature review | Single | Dedicated pharmacist  Audit  Feedback  Pharmacist on rounds  Education and training  Pharmacist champion | Element 81%-100%  Bundle 54% | Pathogen eradication with and without stewardship pharmacist  98% v 90% p=0.09 |

Candida bundle

| **Author and year**  Case number  Element number  *Study design* | Bundle development | Single or multi-centre | Implementation  strategies | Compliance measured | Outcome measured |
| --- | --- | --- | --- | --- | --- |
| **Antworth 2013**  37 pre  41 post  6 elements  *Non-parallel cohort study* | AST team identified key elements from Infectious Diseases Society of America’s clinical practice guidelines for canadidaemia | Single | Electronic surveillance and alerting  Education  Web-based patient tracking  Web-based checklist tracking  Antibiotic stewardship team formed (MDT)  AST advising  AST champions | Element 85.4%-100%  Bundle 78% | Length of stay 20 v 21 days p=0.92  Time to clearance of candida  3v3 days p=0.61  pre and intervention group |
| **Takesue 2014**  608 cases  3+6 elements  (immediate & late)  *Prospective cohort study* | ACTIONS project- based on literature review | Multi | Education  Checklist available electronically | Element 24.3%-84.5%  Bundle 6.9% | Clinical success rate (resolution of signs and symptoms of candidaemia) in compliant v non-compliant  92.9% v 75.8% p=0.011 |

COPD bundle

| **Author and year**  Case number  Element number  *Study design* | Bundle development | Single or multi-centre | Implementation  strategies | Compliance measured | Outcome measured |
| --- | --- | --- | --- | --- | --- |
| **Hopkinson 2011**  22 baseline  94 post  6 elements  *Non-parallel cohort study* | National and international guidelines, literature review, MDT project team, patient survey and local meetings | Single | MDT formation  Survey of staff knowledge prior to starting  Education  Posters of algorithm  Weekly MDT meeting to refine delivery  Incentivize nurses to complete checklist  Barriers to implementation addressed  PDSA cycles | Element 39%-100% | Readmission rates in bundle used v not used. (95%CI)  16.4% v 10.8% (-2.1% -13.2%) |
| **McCarthy**  50 pre  51 post  10 elements  *Non-parallel cohort study* | Based on literature review, clinical advisory group and MDT | Single | Bundle printed and attached to patients notes  Education  Presented bundle at clinical meeting | Element 33%-98% | Readmission in 30 days  12% v 14% p=0.75  Mean length of stay  11.8 v 9 days p=0.377  (pre and post implementation) |

C. Diff. bundle

| **Author and year**  Case number  Element number  *Study design* | Bundle development | Single or multi-centre | Implementation  strategies | Compliance measured | Outcome measured |
| --- | --- | --- | --- | --- | --- |
| **Bishop 2013**  39,093 cases  6 elements  *Non-parallel cohort study* | Based on SCIP and hospital developed protocols | Single | Education  Dissemination of antibiotic guidelines  Electronic limiting of antibiotic and PPI prescriptions | Element partial  96%-99% | C.difficile average monthly rates pre and post implementation  4.13 v 1.93 p=0.03 |
| **Dewart 2017**  120 cases (59 bundle used and 61 not used)  3 elements  *Retrospective observational cohort study* | Based on institutions guidelines | Single | Education  Incorporated the bundle into IT system | Element partial  86.4% | Mean time to initiation of contact precautions with bundle use versus without  22.4 hrs v 33.7 hrs p<0.04 |

Discharge bundle

| **Author and year**  Case number  Element number  *Study design* | Bundle development | Single or multi-centre | Implementation  strategies | Compliance measured | Outcome measured |
| --- | --- | --- | --- | --- | --- |
| **Shermont 2016**  Sample of 10 families per week for 16 months  (454 post intervention interviews)  4 elements  *Non-parallel cohort study* | Developed by institution from discharge analysis and PDSA cycles | Single | PDSA cycles  Nursing leadership staff evaluated, coached and supported staff  Teach back method- standardising discharge process  Baseline audit and feedback  MDT,  Assessed barriers  Executive backing  Piloting and scaling up  Assigning roles,  RCA  Aligning with national goals and setting goals  Comprehensive toolbox for dissemination to other units  Education  Champions and superusers | Element all >90% | 7 day readmission rate pre and post implementation 4.18% v 3.81% p<0.05 |
| **Mallory 2017**  2601 cases  4 elements  *Prospective observational cohort study* | Developed by institution based on literature review and American Academy of Pediatric Guidelines | Multi | Local improvement leaders  MDT formation  Promote inter-professional buy-in  Monthly conference calls between 4 pilot sites  PDSA cycles  Integrated elements into EHR  Teach-back training Education  Provided incentives for perfect primary care provider handoff  Arranged interpreters  Identified barriers  Nurse educators appointed to train teach back  Phone call scripts developed | Element 27.7%-77.7% | Patient reuse rate unchanged for both technology and non-technology supported patients 27% and 7.2% respectively |

Emergency laparotomy bundle

| **Author and year**  Case number  Element number  *Study design* | Bundle development | Single or multi-centre | Implementation  strategies | Compliance measured | Outcome measured |
| --- | --- | --- | --- | --- | --- |
| **Huddart 2014**  299 pre  427 post  5 elements  *Non-parallel cohort study* | Based on data from one hospital in ELN and RCS & DoH recommendations | Multi | MDT formation  Executive board acceptance  PDSA cycles  Local resolution of problems  Education  Posters and emails  Feedback to key stakeholders  Address barriers | Element 67%-83% | Mix-adjusted risk of death pre and post implementation (95%CI)  15.6% - 9.6%  RR 0.641 (0.45-0.83)  p=0.002 |

Falls prevention bundle

| **Author and year**  Case number  Element number  *Study design* | Bundle development | Single or multi-centre | Implementation  strategies | Compliance measured | Outcome measured |
| --- | --- | --- | --- | --- | --- |
| **Healey 2013**  baseline= 33,583 OBD, intervention = 33,495 OBD (observed bed days)  9 elements  *Prospective case control study* | Based on Royal college of Physicians fall safe | Multi | Nurse leads  Education and training  Salary up-lift to leads  Phased introduction  Champions  Leads addressing barriers- measuring progress and feedback  Funding for small scale changes | Element 70%-100% | Reported falls rate reduction pre and post implementation intervention (95%CI)  Adjusted rate ratio 0.75 (0.68-0.84) p<0.001 |
| **Richardson 2015**  Total 9,679  Bundle All 7 elements  Bundle vulnerable 14 elements  Bundle post fall 11 elements  *Prospective observational study* | Based on Royal college of Physicians fall safe: adapted and modified | Multi | Monthly audits and feedback  Root Cause Analysis  Training by falls team  Sticker in notes to remind post-fall  Address barriers | Bundle All 65-38%  Bundle vulnerable 0%-11%  Bundle post-fall 5%-17% | Reported falls reduced by 13%  No significance testing |
| **Sutton 2014**  280 Bundle A  Bundle B unclear  11 elements in A  10 elements in B  *Non-parallel cohort study* | Based on Royal college of Physicians fall safe | Single | Senior management support  Project coordinator  Clinical educators  Education and training  Champions  PDSA cycles | Element 50-100% | No outcome stated |

Hip fracture bundle

| **Author and year**  Case number  Element number  *Study design* | Bundle development | Single or multi-centre | Implementation  strategies | Compliance measured | Outcome measured |
| --- | --- | --- | --- | --- | --- |
| **Bandara 2016**  221 pre  198 post  *5 elements*  *Non-parallel cohort study* | Developed by institution based on literature review | Single | Assigning roles  Goals forms available during rounds and MDT meetings  MDT formation  Training in MSQ and CAM forms  Education  Neck of femur nurse champion | Element 67%-92%  Bundle 47% | Compliance with bundle pre and post  0% v 47% (no p value) |

Medication error bundle

| **Author and year**  Case number  Element number  *Study design* | Bundle development | Single or multi-centre | Implementation  strategies | Compliance measured | Outcome measured |
| --- | --- | --- | --- | --- | --- |
| **Hauser 2010**  Unknown  4 elements  *Non-parallel cohort study* | Developed by pharmacy planning and implementation group in hospital | Single | Steering team formed  Survey of competency prior to bundle  Pharmacy management leads  Mandatory signing of bundle sheet  Focus groups  Continuous feedback | Bundle 45%-90% spot checking | Drug errors rate per 1000 patients days pre v post implementation  0.116 v 0.086 p<0.008 |

Meningitis bundle

| **Author and year**  Case number  Element number  *Study design* | Bundle development | Single or multi-centre | Implementation  strategies | Compliance measured | Outcome measured |
| --- | --- | --- | --- | --- | --- |
| **Viale 2015**  92 pre  85 post  3 elements  *Non-parallel cohort study* | Developed by MDT group | Multi | MDT formation  Protocol for transfer  Education and training | Element partial  100% | In-hospital mortality pre and post implementation  14.1% v 4.7% p=0.04 |

MRSA bundle

| **Author and year**  Case number  Element number  *Study design* | Bundle development | Single or multi-centre | Implementation  strategies | Compliance measured | Outcome measured |
| --- | --- | --- | --- | --- | --- |
| **Bessesen 2013**  Unknown  6 elements hospital A  5 elements hospital B  *Prospective cohort study* | Based on CDC precautions- bundle and screening developed in each hospital with institutional change culture | Multi | Education and training  Champions  Feedback monthly to frontline staff  Educational material  Collaboration to resolve barriers  Daily hand hygiene surveillance  Daily compliance with elements checked  Regular observations of barrier precautions | Bundle  Hospital A 94.4%  Hospital B 73.3% | MRSA transmission rates per 1000 patient days  Hospital A 1.58  Hospital B 1.56  p= 0.98 |
| **Kawamura 2016**  1333 pre  1966 post  5 elements  *Non-parallel cohort study* | Developed by institutions infection control team | Single | Manual distributed  Feedback | Element partial  93.6%- 97.4% | MRSA SSI infection rate pre and post intervention  2.18% v 0.97% p=0.003 |

Peripheral cannula bundle

| **Author and year**  Case number  Element number  *Study design* | Bundle development | Single or multi-centre | Implementation  strategies | Compliance measured | Outcome measured |
| --- | --- | --- | --- | --- | --- |
| **Fakih 2012**  4904 cases  4 elements  *Non-parallel cohort controlled crossover study* | Developed in hospital based on institutes metrics | Single | Education and assessment  Posters, pocket cards and brochures  Feedback  Process measures shared monthly  Research nurses  Nurse survey of value of program  Evaluated care and complications for 4 periods and intervened through education and feedback | Element 56%-95% | Infection rate per 10,000 patient days pre and post implementation  2.2 v 0.44 p=0.016 |
| **Mestre 2013**  180 pre  2145 post  3 elements  *Non-parallel cohort study* | Adapted from CDC guidelines | Single | Education  MDT formation  Audit and feedback  Change elements to improve compliance | Element 68.2% | Peripheral vein phlebitis  Pre- 23.3/100 catheter days (95%CI 16.4-30.1)  Post- 12.1/100 catheter days (95%CI 10.7-13.2) |

Staph. Aureus bundle

| **Author and year**  Case number  Element number  *Study design* | Bundle development | Single or multi-centre | Implementation  strategies | Compliance measured | Outcome measured |
| --- | --- | --- | --- | --- | --- |
| **Borde 2014**  39 pre  20 post  5 elements  *Non-parallel cohort study* | Based on internal clinical guidelines focusing on standardized bundle approach to treatment of SA-BSIs | Single | Education  Bedside explanation of bundle | Element 55%-80% | In-hospital death pre and post intervention  43.6% v 10% p=0.009 |
| **Lopéz-Cortes 2013**  287 pre  221 post  6 elements  *Non-parallel cohort study* | Based on SR of literature | Multi | Education  Letters to heads of services  Regular follow-up  Checklist of bundle added to patients chart and rational for intervention | Element 55.6%-91.3% | 30 day mortality pre and intervention period  OR 0.56 (95% CI 0.34-0.93) |
| **Nguyen 2015**  82 pre  88 post  7 elements  *Non-parallel cohort study* | Literature review and aligned with IDSA MRSA quality performance measures | Single | Formation of antibiotic stewardship team (MDT)  Teams and pharmacist notified  Clinical surveillance software  Champion-pharmacist  Education  Introductory launch lecture  Pocket cards for physicians | Element 94%-97.6%  Bundle 84.1% | 30 day readmission  11% v 1.1% p=0.008  Mortality pre and intervention group  19.5% v 11.4% p=0.2 |

Stroke bundle

| **Author and year**  Case number  Element number  *Study design* | Bundle development | Single or multi-centre | Implementation  strategies | Compliance measured | Outcome measured |
| --- | --- | --- | --- | --- | --- |
| **Power 2015**  1068 control  1176 intervention  (random sample assessed)  4 early elements  5 rehab elements  *Randomised cluster trial* | Developed by National Stroke Audit | Multi | Education-multi level  Executive mentor visits  PDSA cycles  Access to project director  Web based improvement advisor  Webinars  Chief executive backing  Web-based submission system | Bundle  Early 27.3%  Rehab 46.2% | Relative increase in odds of compliance of intervention group compared to control group (95% CI)  1.56  (1.06-2.31) p=0.025 |
| **Nakibuuka 2016**  127 pre  127 post  6 elements  *Non-parallel cohort study* | Adopted Melbourne stroke care bundle | Single | Distribution of educational material and protocol  Funding to ensure timely investigations  Sufficient stock and equipment  Distributing assessment tools  Education and training  MDT  Nurse lead  Role assignment  Weekly meetings with senior supervision to monitor adherence  Assessing barriers | Element partial 100% | 30 day mortality rate  RR 1.2 (95% CI 0.5-2.6) |

Tracheostomy bundle

| **Author and year**  Case number  Element number  *Study design* | Bundle development | Single or multi-centre | Implementation  strategies | Compliance measured | Outcome measured |
| --- | --- | --- | --- | --- | --- |
| **Hettige 2008**  24 pre  70 post  7 elements  *Non-parallel cohort study* | Developed by tracheostomy working group in hospital using other hospital guidelines and literature evidence | Single | Bundle on intranet  Education and training  Escalation policy  Tracheostomy working group developed | Element 9%-96%  Bundle 58% | Clinical incidence rate of severe events pre and post implementation  27% v 10% p<0.05 |

Urinary catheter bundle

| **Author and year**  Case number  Element number  *Study design* | Bundle development | Single or multi-centre | Implementation  strategies | Compliance measured | Outcome measured |
| --- | --- | --- | --- | --- | --- |
| **Andreessen 2012**  90 pre  51 post  5 elements  *Non-parallel cohort study* | Adapted from another centres bundle | Single | MTD formation  Education and training  Educational emails  Phased in  Update supplies  Electronic document for bundle  Electronic order template  Posters and hand-out | Element 80%-100% | Mean catheter device days pre and post implementation (SD)  5.6(5.15) v 2.9 (2.23) days  p<0.01 |

Ventilator associated pneumonia bundle

| **Author and year**  Case number  Element number  *Study design* | Bundle development | Single or multi-centre | Implementation  strategies | Compliance measured | Outcome measured |
| --- | --- | --- | --- | --- | --- |
| **De Luca 2017**  192 pre  153 post  7 elements  *Non-parallel cohort study* | IHI VAP bundle | Single  ED | Order sets developed  New ETT for intubation  Staff training  VAP supply carts  Champions  Feedback to nursing staff  Real-time mentoring | Element 3%-68% | Reduction in VAP rates  Hazard ratio 0.26 p=0.005 |

**Appendix 2B**

Quality of included papers scoring

| 1^st^ Author | Year | Country | Bundle | N | Reporting (11) | External validity  (3) | Internal validity- bias  (7) | Internal validity- confounding (6) | Power  (1) | Total  (28) |
| --- | --- | --- | --- | --- | --- | --- | --- | --- | --- | --- |
| Aguirre –Tejedo^1^ | 2009 | Spain | Sepsis | 40 cases | 4 | 3 | 4 | 2 | 0 | **13** |
| Andreessen^2^ | 2012 | USA | Urinary catheter | 90 pre  51 post | 5 | 3 | 5 | 2 | 0 | **15** |
| Antworth^3^ | 2013 | USA | Candidaemia | 37 pre  41 post | 8 | 3 | 5 | 1 | 0 | **17** |
| Apisarnthanark^4^ | 2010 | Thailand | Central line | 155 pre  325 post | 9 | 3 | 5 | 1 | 0 | **18** |
| Baldwin^5^ | 2008 | UK | Sepsis | 32 cases | 5 | 3 | 4 | 1 | 0 | **13** |
| Bandara^6^ | 2016 | Australia | Hip fracture | 221 pre  198 post | 7 | 3 | 5 | 2 | 0 | **17** |
| Berg^7^ | 2013 | USA | Sepsis | 123 cases | 8 | 3 | 4 | 3 | 0 | **18** |
| Bessesen^8^ | 2013 | USA | MRSA | Unknown | 5 | 3 | 5 | 1 | 0 | **14** |
| Bishop^9^ | 2013 | USA | C. Difficile | 39,093 cases | 5 | 3 | 5 | 1 | 0 | **14** |
| Borde^10^ | 2014 | Germany | Staph. Aureus bacteraemia | 39 pre  20 post | 7 | 3 | 5 | 1 | 0 | **16** |
| Bruce^11^ | 2015 | USA | Sepsis | 62 pre  133 post | 8 | 3 | 5 | 2 | 0 | **18** |
| Brumley^12^ | 2016 | USA | C. Difficile | 83 baseline  83 post | 8 | 3 | 5 | 2 | 1 | **19** |
| Bull^13^ | 2011 | Australia | SSI | 180 pre  275 post | 5 | 2 | 5 | 1 | 0 | **13** |
| Bundy^14^ | 2014 | USA | Central line | Unknown | 6 | 1 | 4 | 1 | 0 | **12** |
| Carreno^15^ | 2015 | USA | Antimicrobial stewardship | 162 pre  124 post | 8 | 3 | 5 | 1 | 1 | **18** |
| Casserly^16^ | 2011 | USA | Sepsis | 87 cases | 7 | 3 | 5 | 3 | 0 | **18** |
| Choi^17^ | 2013 | USA | Central line | 235 pre  221 post | 5 | 2 | 5 | 1 | 0 | **13** |
| Crolla^18^ | 2012 | USA | SSI | 1537 cases (Compliance measured on 10 patients quarterly from June ‘09-Oct ’11) | 6 | 3 | 5 | 2 | 0 | **16** |
| Coba^19^ | 2011 | USA | Sepsis | 498 cases | 8 | 3 | 5 | 3 | 0 | **19** |
| Daniels^20^ | 2011 | UK | Sepsis | 567 cases | 9 | 3 | 5 | 2 | 0 | **19** |
| De Luca^21^ | 2017 | USA | Ventilator | 192 pre  153 post | 8 | 2 | 6 | 2 | 1 | **19** |
| De Miguel-Yanes^22^ | 2009 | Spain | Sepsis | 53 pre  50 post | 8 | 3 | 5 | 2 | 0 | **18** |
| Dewart^23^ | 2017 | USA | C. Difficile | 120 cases | 7 | 3 | 5 | 3 | 0 | **18** |
| Duffy^24^ | 2015 | USA | Central line | 35 pre  45 post (random sample) | 5 | 3 | 5 | 1 | 0 | **14** |
| Dumyati^25^ | 2014 | USA | Central line | Unknown | 6 | 1 | 5 | 1 | 0 | **13** |
| Fakih^26^ | 2012 | USA | Peripheral cannula | 4904 cases | 5 | 3 | 5 | 1 | 0 | **14** |
| Ferrer^27^ | 2008 | Spain | Sepsis | 854 pre  1465 post | 9 | 3 | 5 | 2 | 0 | **19** |
| Fisher^28^ | 2016 | USA | SSI | 545 pre  324 post | 5 | 2 | 4 | 1 | 0 | **12** |
| Flynn^29^ | 2015 | USA | Sepsis | 59 pre  49 post | 9 | 3 | 5 | 1 | 0 | **18** |
| Forbes^30^ | 2008 | Canada | SSI | 105 pre  103 post | 9 | 3 | 5 | 1 | 1 | **19** |
| Freixas^31^ | 2012 | Spain | Central and peripheral line | Unknown | 7 | 3 | 4 | 2 | 0 | **16** |
| Girardis^32^ | 2009 | Italy | Sepsis | 67 cases | 9 | 3 | 5 | 2 | 0 | **19** |
| Gould^33^ | 2016 | USA | SSI | 88 pre  126 post | 5 | 2 | 5 | 1 | 0 | **13** |
| Grigonis^34^ | 2017 | USA | CVC | 6660 pre  6559 post | 7 | 2 | 5 | 1 | 0 | **15** |
| Hauser^35^ | 2010 | USA | Medication errors | Unknown | 5 | 1 | 4 | 2 | 0 | **12** |
| Healey^36^ | 2013 | UK | Falls prevention | Unknown | 5 | 3 | 5 | 2 | 0 | **15** |
| Hedrick^37^ | 2007 | USA | SSI | 379 baseline  390 post | 9 | 3 | 5 | 2 | 0 | **19** |
| Hettige^38^ | 2008 | UK | Tracheostomy care | 24 pre  70 post | 4 | 3 | 4 | 1 | 0 | **12** |
| Hopkinson^39^ | 2011 | UK | COPD | 22 baseline  94 post | 5 | 3 | 5 | 1 | 0 | **14** |
| Huddart^40^ | 2014 | UK | Emergency laparotomy | 299 pre  427 post | 9 | 3 | 5 | 2 | 0 | **19** |
| Jaggi^41^ | 2014 | India | Central line | Unknown | 5 | 0 | 5 | 1 | 0 | **11** |
| Jeon^42^ | 2013 | South Korea | Sepsis | 163 pre  203 post | 9 | 3 | 5 | 2 | 0 | **19** |
| Joslin^43^ | 2015 | UK | Acute kidney injury | 100 pre  92 post | 8 | 3 | 3 | 1 | 0 | **15** |
| Kakebeeke^44^ | 2013 | Netherlands | Sepsis | 323 cases | 9 | 3 | 5 | 1 | 0 | **18** |
| Kalich^45^ | 2016 | USA | Sepsis | 62 pre  62 post | 9 | 3 | 5 | 1 | 0 | **18** |
| Kang^46^ | 2012 | South Korea | Sepsis | 317 cases | 9 | 3 | 5 | 3 | 0 | **20** |
| Kawamura^47^ | 2016 | Japan | MRSA prevention | 1333pre  1966 post | 7 | 2 | 5 | 1 | 0 | **15** |
| Kim^48^ | 2014 | South Korea | Sepsis | 88 pre  87 post | 9 | 3 | 5 | 1 | 0 | **18** |
| Kleidon^49^ | 2014 | Australia | Central line | 42 pre  50 post | 6 | 3 | 5 | 3 | 0 | **17** |
| Kolhe^50^ | 2015 | UK | Acute kidney injury | 1209 pre  1291 post | 9 | 3 | 5 | 3 | 0 | **20** |
| Kolhe^51^ | 2016 | UK | Acute kidney injury | 3351 patients | 9 | 3 | 5 | 3 | 0 | **20** |
| Kuan^52^ | 2013 | Singapore | Sepsis | 117 cases | 8 | 3 | 4 | 2 | 0 | **17** |
| Laguna-Perez^53^ | 2012 | Spain | Sepsis | 84 pre  41 post | 8 | 3 | 5 | 2 | 0 | **18** |
| Larochelle^54^ | 2011 | USA | SSI | 706 cases | 5 | 3 | 4 | 2 | 0 | **14** |
| Levy^55^ | 2010 | Worldwide | Sepsis | 15,022 cases | 7 | 3 | 5 | 2 | 0 | **17** |
| Liau^56^ | 2010 | Singapore | SSI | 1040 pre  2408 post | 5 | 3 | 4 | 1 | 0 | **13** |
| Lippitt^57^ | 2017 | USA | SSI | 91 pre  128 post | 9 | 2 | 5 | 1 | 0 | **17** |
| Liu^58^ | 2016 | USA | Sepsis | 5942 pre  6544 post | 8 | 3 | 5 | 2 | 0 | **18** |
| Lopez-Cortes^59^ | 2013 | Spain | Staph. Aureus bacteraemia | 287 pre  221 post | 9 | 3 | 5 | 2 | 0 | **19** |
| MacRedmond^60^ | 2010 | Canada | Sepsis | 37 pre  37 post | 9 | 3 | 5 | 1 | 0 | **18** |
| McCarthy^61^ | 2013 | Ireland | COPD | 50 pre  51 post | 8 | 3 | 5 | 1 | 0 | **17** |
| Mallory^62^ | 2016 | USA | Discharge | 2601 cases | 5 | 2 | 5 | 0 | 1 | **13** |
| Mestre^63^ | 2013 | Spain | Peripheral cannula | 180 pre  2145 post | 7 | 3 | 5 | 2 | 0 | **17** |
| Micek^64^ | 2006 | USA | Sepsis | 60 pre  60 post | 7 | 3 | 5 | 2 | 0 | **17** |
| Nakibuuka^65^ | 2016 | Uganda | Stroke | 127 pre  127 post | 10 | 2 | 5 | 3 | 1 | **21** |
| Nguyen C^66^ | 2015 | USA | Staph. Aureus bacteraemia | 82 pre  88 post | 8 | 3 | 5 | 2 | 0 | **18** |
| Nguyen H^67^ | 2007 | USA | Sepsis | 330 cases | 8 | 3 | 4 | 3 | 0 | **18** |
| Nguyen H^68^ | 2011 | Asia | Sepsis | 556 cases | 9 | 3 | 3 | 3 | 0 | **18** |
| Noritomi^69^ | 2014 | Brazil | Sepsis | 203 baseline  1917 post | 9 | 3 | 4 | 2 | 0 | **18** |
| Palleschi^70^ | 2014 | USA | Sepsis | 50 baseline  100 post | 5 | 3 | 4 | 1 | 0 | **13** |
| Pastor^71^ | 2010 | USA | SSI | 491 cases | 8 | 3 | 5 | 1 | 0 | **17** |
| Perez-Blanco^72^ | 2015 | Spain | SSI | 218 pre  124 post | 8 | 3 | 5 | 1 | 0 | **17** |
| Plambech^73^ | 2012 | Denmark | Sepsis | 27 baseline  77 post | 5 | 3 | 5 | 1 | 0 | **14** |
| Power^74^ | 2014 | UK | Stroke | 1068 controls  1176 intervention (random sample assessed) | 9 | 3 | 5 | 2 | 0 | **19** |
| Ramsdell^75^ | 2017 | USA | Sepsis | 48 pre  110 post | 7 | 3 | 5 | 1 | 0 | **16** |
| Richardson^76^ | 2015 | UK | Falls prevention | 9679 patient episodes | 3 | 3 | 4 | 2 | 0 | **12** |
| Rinke^77^ | 2012 | USA | Central line | Unknown | 9 | 2 | 5 | 1 | 0 | **17** |
| Ryckman^78^ | 2009 | USA | SSI | Unknown | 3 | 0 | 5 | 1 | 0 | **9** |
| Schiavone^79^ | 2017 | USA | SSI | 115 pre  118 post | 8 | 3 | 4 | 1 | 0 | **16** |
| Schriefer^80^ | 2017 | USA | SSI | 154 pre  387 post | 4 | 2 | 4 | 1 | 0 | **11** |
| Secola^81^ | 2012 | USA | Central line | 41 no intervention  41 intervention | 8 | 3 | 5 | 4 | 0 | **20** |
| Seoane^82^ | 2013 | USA | Sepsis | 1105 cases | 4 | 3 | 5 | 1 | 0 | **13** |
| Shermont^83^ | 2016 | USA | Discharge | Unclear | 5 | 1 | 5 | 2 | 0 | **13** |
| Shin^84^ | 2013 | South Korea | Sepsis | 770 cases | 9 | 3 | 5 | 3 | 0 | **20** |
| Sutton^85^ | 2014 | UK | Falls prevention | Unclear | 3 | 2 | 3 | 0 | 0 | **8** |
| Takesue^86^ | 2014 | Japan | Candiaemia | 608 cases | 6 | 2 | 5 | 3 | 0 | **16** |
| Tanner^87^ | 2016 | UK | SSI | 127 pre  166 post | 8 | 3 | 5 | 2 | 0 | **18** |
| Tillman^88^ | 2013 | USA | SSI | Unknown snapshot | 5 | 3 | 4 | 1 | 0 | **13** |
| Toltzis^89^ | 2014 | USA | SSI | Unknown | 5 | 3 | 5 | 1 | 0 | **14** |
| Toth^90^ | 2010 | USA | Antibiotic stewardship | 80 pre  80 post | 7 | 3 | 4 | 1 | 0 | **15** |
| Tromp^91^ | 2010 | Netherlands | Sepsis | 159 pre  666 post | 7 | 3 | 5 | 1 | 0 | **16** |
| Van der Slegt^92^ | 2013 | Netherlands | SSI | 720 cases  (10 random procedures observed quarterly) | 8 | 3 | 4 | 2 | 0 | **17** |
| Viale^93^ | 2015 | Italy | Meningitis | 92 pre  85 post | 9 | 3 | 5 | 1 | 0 | **18** |
| Wang^94^ | 2013 | China | Sepsis | 78 pre  117 post | 7 | 3 | 4 | 1 | 0 | **15** |
| Westphal^95^ | 2011 | Brazil | Sepsis | 102 pre  115 post | 9 | 3 | 5 | 2 | 0 | **19** |
| Wheeler^96^ | 2011 | USA | Central line | Unknown | 4 | 3 | 5 | 1 | 0 | **13** |
| Whippy^97^ | 2011 | USA | Sepsis | Unknown | 4 | 3 | 5 | 1 | 0 | **13** |
| Wick^98^ | 2008 | USA | SSI | 298 cases | 8 | 3 | 5 | 1 | 0 | **17** |
| Wick^99^ | 2012 | USA | SSI | 278 baseline  324 cases | 8 | 3 | 5 | 2 | 0 | **18** |

References

1. Aguirre Tejedo A, Echarte Pazos JL, Minguez Maso S, Supervia Caparros A, Skaf Peters E, Campodarve Botet I. Emergency department implementation of a severe sepsis code. *EMERGENCIAS*. 2009;21(4):255-261. http://apps.webofknowledge.com/full_record.do?product=UA&search_mode=GeneralSearch&qid=1&SID=S1gTppbKymHDwA45Zdd&page=1&doc=5. Accessed December 4, 2015.

2. Andreessen L, Wilde MH, Herendeen P. Preventing catheter-associated urinary tract infections in acute care: the bundle approach. *J Nurs Care Qual*. 2012;27(3):209-217. doi:10.1097/NCQ.0b013e318248b0b1.

3. Antworth A, Collins CD, Kunapuli A, et al. Impact of an antimicrobial stewardship program comprehensive care bundle on management of candidemia. *Pharmacotherapy*. 2013;33(2):137-143. doi:10.1002/phar.1186.

4. Apisarnthanarak A, Thongphubeth K, Yuekyen C, Warren DK, Fraser VJ. Effectiveness of a catheter-associated bloodstream infection bundle in a Thai tertiary care center: A 3-year study. *Am J Infect Control*. 2010;38(6):449-455. doi:10.1016/j.ajic.2009.08.017.

5. Baldwin LN, Smith SA, Fender V, Gisby S, Fraser J. An audit of compliance with the sepsis resuscitation care bundle in patients admitted to A&amp;E with severe sepsis or septic shock. *Int Emerg Nurs*. 2008;16(4):250-256. doi:10.1016/j.ienj.2008.05.008.

6. Bandara S, Lynch G, Cooke C, Varghese P, Ward N. Using Care Bundles to Improve Surgical Outcomes and Reduce Variation in Care for Fragility Hip Fracture Patients. *Geriatr Orthop Surg Rehabil*. 2017;8(2):104-108. doi:10.1177/2151458516681634.

7. Berg GM, Vasquez DG, Hale LS, Nyberg SM, Moran DA. Evaluation of Process Variations in Noncompliance in the Implementation of Evidence-Based Sepsis Care. *J Healthc Qual*. 2013;35(1):60-69. doi:10.1111/j.1945-1474.2011.00168.x.

8. Bessesen MT, Lopez K, Guerin K, et al. Comparison of control strategies for methicillin-resistant Staphylococcus aureus. *Am J Infect Control*. 2013;41(11):1048-1052. doi:10.1016/j.ajic.2013.01.032.

9. Bishop J, Parry MF, Hall T. Decreasing Clostridium difficile infections in surgery: impact of a practice bundle incorporating a resident rounding protocol. *Conn Med*. 2013;77(2):69-75. http://www.ncbi.nlm.nih.gov/pubmed/23513633.

10. Borde JP, Batin N, Rieg S, et al. Adherence to an antibiotic stewardship bundle targeting Staphylococcus aureus blood stream infections at a 200-bed community hospital. *Infection*. 2014;42(4):713-719. doi:10.1007/s15010-014-0633-1.

11. Bruce HR, Maiden J, Fedullo PF, Kim SC. Impact of Nurse-Initiated ED Sepsis Protocol on Compliance With Sepsis Bundles, Time to Initial Antibiotic Administration, and In-Hospital Mortality. *J Emerg Nurs*. 2015;41(2):130-137. doi:10.1016/j.jen.2014.12.007.

12. Brumley PE, Malani AN, Kabara JJ, Pisani J, Collins CD. Effect of an antimicrobial stewardship bundle for patients with Clostridium difficile infection. *J Antimicrob Chemother*. 2016;71(3):836-840. doi:10.1093/jac/dkv404.

13. Bull A, Wilson J, Worth LJ, et al. A bundle of care to reduce colorectal surgical infections: An Australian experience. *J Hosp Infect*. 2011;78(4):297-301. doi:10.1016/j.jhin.2011.03.029.

14. Bundy DG, Gaur AH, Billett AL, He B, Colantuoni EA, Miller MR. Preventing CLABSIs among pediatric hematology/oncology inpatients: national collaborative results. *Pediatrics*. 2014;134(6):e1678-85. doi:10.1542/peds.2014-0582.

15. Carreno JJ, Kenney RM, Bloome M, et al. Evaluation of pharmacy generalists performing antimicrobial stewardship services. *Am J Heal Pharm*. 2015;72(15):1298-1303. doi:10.2146/ajhp140619.

16. Casserly B, Baram M, Walsh P, Sucov A, Ward NS, Levy MM. Implementing a collaborative protocol in a sepsis intervention program: Lessons learned. *Lung*. 2011;189(1):11-19. doi:10.1007/s00408-010-9266-z.

17. Choi SW, Chang L, Hanauer DA, et al. Rapid reduction of central line infections in hospitalized pediatric oncology patients through simple quality improvement methods. *Pediatr Blood Cancer*. 2013;60(2):262-269. doi:10.1002/pbc.24187.

18. Crolla RMPH, van der Laan L, Veen EJ, Hendriks Y, van Schendel C, Kluytmans J. Reduction of surgical site infections after implementation of a bundle of care. *PLoS One*. 2012;7(9):e44599. doi:10.1371/journal.pone.0044599.

19. Coba V, Whitmill M, Mooney R, et al. Resuscitation bundle compliance in severe sepsis and septic shock: improves survival, is better late than never. *J Intensive Care Med*. 2011;26(5):304-313. doi:10.1177/0885066610392499.

20. Daniels R, Nutbeam T, McNamara G, Galvin C. The sepsis six and the severe sepsis resuscitation bundle: a prospective observational cohort study. *Emerg Med J*. 2011;28(6):507-512. doi:10.1136/emj.2010.095067.

21. DeLuca LA, Walsh P, Davidson DD, et al. Impact and feasibility of an emergency department–based ventilator-associated pneumonia bundle for patients intubated in an academic emergency department. *Am J Infect Control*. 2017;45(2):151-157. doi:10.1016/j.ajic.2016.05.037.

22. De Miguel-Yanes JM, Muñoz-González J, Andueza-Lillo J a., Moyano-Villaseca B, González-Ramallo VJ, Bustamante-Fermosel A. Implementation of a bundle of actions to improve adherence to the Surviving Sepsis Campaign guidelines at the ED. *Am J Emerg Med*. 2009;27(6):668-674. doi:10.1016/j.ajem.2008.05.010.

23. Dewart CM, Blanco N, Foxman B, Malani AN. Electronic clostridium difficile infection bundle reduces time to initiation of contact precautions. In: *Infection Control and Hospital Epidemiology*. Vol 38. ; 2017:242-244. doi:10.1017/ice.2016.250.

24. Duffy EA, Rodgers CC, Shever LL, Hockenberry MJ. Implementing a Daily Maintenance Care Bundle to Prevent Central Line-Associated Bloodstream Infections in Pediatric Oncology Patients. *J Pediatr Oncol Nurs*. 2015;(4):1-8. doi:10.1177/1043454214563756.

25. Dumyati G, Concannon C, van Wijngaarden E, et al. Sustained reduction of central line–associated bloodstream infections outside the intensive care unit with a multimodal intervention focusing on central line maintenance. *Am J Infect Control*. 2014;42(7):723-730. doi:10.1016/j.ajic.2014.03.353.

26. Fakih MG, Jones K, Rey JE, et al. Sustained Improvements in Peripheral Venous Catheter Care in Non–Intensive Care Units: A Quasi-Experimental Controlled Study of Education and Feedback. *Infect Control Hosp Epidemiol*. 2012;33(5):449-455. doi:10.1086/665322.

27. Ferrer R. Improvement in Process of Care and Outcome After a Multicenter Severe Sepsis Educational Program in Spain. *JAMA*. 2008;299(19):2294. doi:10.1001/jama.299.19.2294.

28. Fisher JC, Godfried DH, Lighter-Fisher J, et al. A novel approach to leveraging electronic health record data to enhance pediatric surgical quality improvement bundle process compliance. *J Pediatr Surg*. 2016;51(6):1030-1033. doi:10.1016/j.jpedsurg.2016.02.080.

29. Flynn JD, McConeghy KW, Flannery AH, Nestor M, Branson P, Hatton KW. Utilization of Pharmacist Responders as a Component of a Multidisciplinary Sepsis Bundle. *Ann Pharmacother*. 2014;48(9):1145-1151. doi:10.1177/1060028014538773.

30. Forbes SS, Stephen WJ, Harper WL, et al. Implementation of Evidence-Based Practices for Surgical Site Infection Prophylaxis: Results of a Pre- and Postintervention Study. *J Am Coll Surg*. 2008;207(3):336-341. doi:10.1016/j.jamcollsurg.2008.03.014.

31. Freixas N, Bella F, Limón E, Pujol M, Almirante B, Gudiol F. Impact of a multimodal intervention to reduce bloodstream infections related to vascular catheters in non-ICU wards: a multicentre study. *Clin Microbiol Infect*. 2013;19(9):838-844. doi:10.1111/1469-0691.12049.

32. Girardis M, Rinaldi L, Donno L, et al. Effects on management and outcome of severe sepsis and septic shock patients admitted to the intensive care unit after implementation of a sepsis program: a pilot study. *Crit Care*. 2009;13(5):R143. doi:10.1186/cc8029.

33. Gould JM, Hennessey P, Kiernan A, Safier S, Herman M. A novel prevention bundle to reduce surgical site infections in pediatric spinal fusion patients. *Infect Control Hosp Epidemiol*. 2016;37(5):527-534. doi:10.1017/ice.2015.350.

34. Grigonis AM, Dawson AM, Burkett M, et al. Use of a Central Catheter Maintenance Bundle in Long-Term Acute Care Hospitals. *Am J Crit Care*. 2016;25(2):165-172. doi:10.4037/ajcc2016894.

35. Hauser DG, Young DA, Braitman LE. Adapting the Bundles Approach to Reduce Medication Errors in Pharmacy Practice. *JCOM March 2010 Vol 17, No 3*. 2010;17(3):125-131.

36. Healey F, Lowe D, Darowski A, et al. Falls prevention in hospitals and mental health units: an extended evaluation of the FallSafe quality improvement project. *Age Ageing*. 2014;43(4):484-491. doi:10.1093/ageing/aft190.

37. Hedrick TL, Turrentine FE, Smith RL, et al. Single-institutional experience with the surgical infection prevention project in intra-abdominal surgery. *Surg Infect (Larchmt)*. 2007;8(4):425-435. doi:10.1089/sur.2006.043.

38. Hettige R, Arora A, Ifeacho S, Narula A. Improving tracheostomy management through design, implementation and prospective audit of a care bundle: how we do it. *Clin Otolaryngol*. 2008;33(5):488-491. doi:10.1111/j.1749-4486.2008.01725.x.

39. Hopkinson NS, Englebretsen C, Cooley N, et al. Designing and implementing a COPD discharge care bundle. *Thorax*. 2012;67(1):90-92. doi:10.1136/thoraxjnl-2011-200233.

40. Huddart S, Peden CJ, Swart M, et al. Use of a pathway quality improvement care bundle to reduce mortality after emergency laparotomy. *Br J Surg*. 2015;102(1):57-66. doi:10.1002/bjs.9658.

41. Jaggi N, Sissodia P. Repeated multimodal supervision programs to reduce the central line-associated bloodstream infection rates in an Indian corporate hospital. *Healthc Infect*. 2014;19(2):53. doi:10.1071/HI13030.

42. Jeon K, Shin TG, Sim MS, et al. Improvements in Compliance WITH Resuscitation Bundles and Achievement of End Points After an Educational Program on the Management of Severe Sepsis and Septic Shock. *Shock*. 2012;37(5):463-467. doi:10.1097/SHK.0b013e31824c31d1.

43. Joslin J, Wilson H, Zubli D, et al. Recognition and management of acute kidney injury in hospitalised patients can be partially improved with the use of a care bundle. *Clin Med (Northfield Il)*. 2015;15(5):431-436. doi:10.7861/clinmedicine.15-5-431.

44. Kakebeeke D, Vis A, de Deckere ER, Sandel MH, de Groot B. Lack of clinically evident signs of organ failure affects ED treatment of patients with severe sepsis. *Int J Emerg Med*. 2013;6(1):4. doi:10.1186/1865-1380-6-4.

45. Kalich BA, Maguire JM, Campbell-Bright SL, et al. Impact of an Antibiotic-specific Sepsis Bundle on Appropriate and Timely Antibiotic Administration for Severe Sepsis in the Emergency Department. *J Emerg Med*. 2016;50(1):79-88.e1. doi:10.1016/j.jemermed.2015.09.007.

46. Kang MJ, Shin TG, Jo IJ, et al. Factors influencing compliance with early resuscitation bundle in the management of severe sepsis and septic shock. *Shock*. 2012;38(5):474-479. doi:10.1097/SHK.0b013e31826eea2b.

47. Kawamura H, Matsumoto K, Shigemi A, et al. A bundle that includes active surveillance, contact precaution for carriers, and cefazolin-based antimicrobial prophylaxis prevents methicillin-resistant Staphylococcus aureus infections in clean orthopedic surgery. *Am J Infect Control*. 2016;44(2):210-214. doi:10.1016/j.ajic.2015.09.014.

48. Kim J, Na S, Yoo YC, Koh SO. Implementing a Sepsis Resuscitation Bundle Improved Clinical Outcome: A Before-and-After Study. *Korean J Crit Care Med*. 2014;29(4):250-256. doi:10.4266/kjccm.2014.29.4.250.

49. Kleidon T, Illing A, Fogarty G, Edwards R, Tomlinson J, Ullman A. Improving the central venous access devices maintenance process to reduce associated infections in paediatrics: evaluation of a practical, multi-faceted quality-improvement initiative. *Healthc Infect*. 2015;20(2):46. doi:10.1071/HI14038.

50. Kolhe N V, Staples D, Reilly T, et al. Impact of Compliance with a Care Bundle on Acute Kidney Injury Outcomes: A Prospective Observational Study. *PLoS One*. 2015;10(7):e0132279. doi:10.1371/journal.pone.0132279.

51. Kolhe N V., Reilly T, Leung J, et al. A simple care bundle for use in acute kidney injury: A propensity score-matched cohort study. *Nephrol Dial Transplant*. 2016;31(11):1846-1854. doi:10.1093/ndt/gfw087.

52. Kuan WS, Mahadevan M, Tan JH, Guo J, Ibrahim I. Feasibility of introduction and implementation of the Surviving Sepsis Campaign bundle in a Singapore Emergency Department. *Eur J Emerg Med*. 2013;20(5):344-349. doi:10.1097/MEJ.0b013e32835c2ba3.

53. Laguna-Pérez A, Chilet-Rosell E, Delgado Lacosta M, Alvarez-Dardet C, Uris Selles J, Muñoz-Mendoza CL. Clinical pathway intervention compliance and effectiveness when used in the treatment of patients with severe sepsis and septic shock at an Intensive Care Unit in Spain. *Rev Lat Am Enfermagem*. 2012;20(4):635-643. http://www.ncbi.nlm.nih.gov/pubmed/22990147.

54. Larochelle M, Hyman N, Gruppi L, Osler T. Diminishing Surgical Site Infections After Colorectal Surgery With Surgical Care Improvement Project: Is It Time to Move on? *Dis Colon Rectum*. 2011;54(4):394-400. doi:10.1007/DCR.0b013e318206165b.

55. Levy MM, Dellinger RP, Townsend SR, et al. The surviving sepsis campaign: Results of an international guideline-based performance improvement program targeting severe sepsis. *Intensive Care Med*. 2010;36(2):222-231. doi:10.1007/s00134-009-1738-3.

56. Liau K-H, Aung K-T, Chua N, et al. Outcome of a strategy to reduce surgical site infection in a tertiary-care hospital. *Surg Infect (Larchmt)*. 2010;11(2):151-159. doi:10.1089/sur.2008.081.

57. Lippitt MH, Fairbairn MG, Matsuno R, et al. Outcomes associated with a five-point surgical site infection prevention bundle in women undergoing surgery for ovarian cancer. In: *Obstetrics and Gynecology*. Vol 130. ; 2017:756-764. doi:10.1097/AOG.0000000000002213.

58. Liu VX, Morehouse JW, Marelich GP, et al. Multicenter implementation of a treatment bundle for patients with sepsis and intermediate lactate values. *Am J Respir Crit Care Med*. 2016;193(11):1264-1270. doi:10.1164/rccm.201507-1489OC.

59. López-Cortés LE, Del Toro MD, Gálvez-Acebal J, et al. Impact of an evidence-based bundle intervention in the quality-of-care management and outcome of Staphylococcus aureus bacteremia. *Clin Infect Dis*. 2013;57(9):1225-1233. doi:10.1093/cid/cit499.

60. MacRedmond R, Hollohan K, Stenstrom R, Nebre R, Jaswal D, Dodek P. Introduction of a comprehensive management protocol for severe sepsis is associated with sustained improvements in timeliness of care and survival. *BMJ Qual Saf*. 2010;19(5):e46-e46. doi:10.1136/qshc.2009.033407.

61. McCarthy C, Brennan JR, Brown L, et al. Use of a care bundle in the emergency department for acute exacerbations of chronic obstructive pulmonary disease: a feasibility study. *Int J Chron Obstruct Pulmon Dis*. 2013;8:605-611. doi:10.2147/COPD.S52883.

62. Mallory LA, Osorio SN, Prato BS, et al. Project IMPACT Pilot Report: Feasibility of Implementing a Hospital-to-Home Transition Bundle. *Pediatrics*. 2017;139(3):e20154626. doi:10.1542/peds.2015-4626.

63. Mestre G, Berbel C, Tortajada P, et al. Successful multifaceted intervention aimed to reduce short peripheral venous catheter-related adverse events: A quasiexperimental cohort study. *Am J Infect Control*. 2013;41(6):520-526. doi:10.1016/j.ajic.2012.07.014.

64. Micek ST, Roubinian N, Heuring T, et al. Before-after study of a standardized hospital order set for the management of septic shock. *Crit Care Med*. 2006;34(11):2707-2713. doi:10.1097/01.CCM.0000241151.25426.D7.

65. Nakibuuka J, Sajatovic M, Nankabirwa J, et al. Effect of a 72 Hour Stroke Care Bundle on Early Outcomes after Acute Stroke: A Non Randomised Controlled Study. *PLoS One*. 2016;11(5):e0154333. doi:10.1371/journal.pone.0154333.

66. Nguyen CT, Gandhi T, Chenoweth C, et al. Impact of an antimicrobial stewardship-led intervention for Staphylococcus aureus bacteraemia: a quasi-experimental study. *J Antimicrob Chemother*. 2015;70(12):3390-3396. doi:10.1093/jac/dkv256.

67. Nguyen HB, Lynch EL, Mou JA, Lyon K, Wittlake WA, Corbett SW. The utility of a quality improvement bundle in bridging the gap between research and standard care in the management of severe sepsis and septic shock in the emergency department. *Acad Emerg Med*. 2007;14(11):1079-1086. doi:10.1197/j.aem.2007.06.024.

68. Nguyen HB, Kuan W, Batech M, et al. Outcome effectiveness of the severe sepsis resuscitation bundle with addition of lactate clearance as a bundle item: a multi-national evaluation. *Crit Care*. 2011;15(5):R229. doi:10.1186/cc10469.

69. Noritomi DT, Ranzani OT, Monteiro MB, et al. Implementation of a multifaceted sepsis education program in an emerging country setting: clinical outcomes and cost-effectiveness in a long-term follow-up study. *Intensive Care Med*. 2014;40(2):182-191. doi:10.1007/s00134-013-3131-5.

70. Palleschi MT eresa, Sirianni S, O’Connor N, Dunn D, Hasenau SM. An interprofessional process to improve early identification and treatment for sepsis. *J Healthc Qual*. 2014;36(4):23-31. doi:10.1111/jhq.12006.

71. Pastor C, Artinyan A, Varma MG, Kim E, Gibbs L, Garcia-Aguilar J. An Increase in Compliance With the Surgical Care Improvement Project Measures Does Not Prevent Surgical Site Infection in Colorectal Surgery. *Dis Colon Rectum*. 2010;53(1):24-30. doi:10.1007/DCR.0b013e3181ba782a.

72. Pérez-Blanco V, García-Olmo D, Maseda-Garrido E, Nájera-Santos MC, García-Caballero J. Evaluación de un paquete de medidas para la prevención de la infección de localización quirúrgica en cirugía colorrectal. *Cirugía Española*. 2015;93(4):222-228. doi:10.1016/j.ciresp.2014.12.003.

73. Plambech MZ, Lurie AI, Ipsen HL. Initial, successful implementation of sepsis guidelines in an emergency department. *Dan Med J*. 2012;59(12).

74. Power M, Tyrrell PJ, Rudd AG, et al. Did a quality improvement collaborative make stroke care better? A cluster randomized trial. *Implement Sci*. 2014;9(1):40. doi:10.1186/1748-5908-9-40.

75. Ramsdell TH, Smith AN, Kerkhove E. Compliance with Updated Sepsis Bundles to Meet New Sepsis Core Measure in a Tertiary Care Hospital. *Hosp Pharm*. 2017;52(3):177-186. doi:10.1310/hpj5203-177.

76. Richardson DA, Bhagwat A, Forster K, et al. The Royal College of Physicians’ Fallsafe care bundles applied trustwide: The Northumbria experience 2013. *Clin Med J R Coll Physicians London*. 2015;15(6):530-535. doi:10.7861/clinmedicine.15-6-530.

77. Rinke ML, Chen AR, Bundy DG, et al. Implementation of a Central Line Maintenance Care Bundle in Hospitalized Pediatric Oncology Patients. *Pediatrics*. 2012;130(4):e996-e1004. doi:10.1542/peds.2012-0295.

78. Ryckman FC, Schoettker PJ, Hays KR, et al. Reducing surgical site infections at a pediatric academic medical center. *Jt Comm J Qual Patient Saf*. 2009;35(4):192-198. http://www.ncbi.nlm.nih.gov/pubmed/19435158.

79. M.B. S, L. M, K. L, et al. Surgical site infection reduction bundle in patients with gynecologic cancer undergoing colon surgery. *Gynecol Oncol*. 2017;147(1):115-119. doi:http://dx.doi.org/10.1016/j.ygyno.2017.07.010.

80. Schriefer J, Sanders J, Michels J, Wolcott K, Ruddy C, Hanson J. Implementation of a Pediatric Orthopaedic Bundle to Reduce Surgical Site Infections. *Orthop Nurs*. 2017;36(1):49-59. doi:10.1097/NOR.0000000000000312.

81. Secola R, Lewis MA, Pike N, Needleman J, Doering L. Feasibility of the use of a reliable and valid central venous catheter blood draw bundle checklist. *J Nurs Care Qual*. 2012;27(3):218-225. doi:10.1097/NCQ.0b013e3182461fab.

82. Seoane L, Winterbottom F, Nash T, et al. Using quality improvement principles to improve the care of patients with severe sepsis and septic shock. *Ochsner J*. 2013;13(3):359-366. http://www.ncbi.nlm.nih.gov/pubmed/24052765.

83. Shermont H, Pignataro S, Humphrey K, Bukoye B. Reducing Pediatric Readmissions. *J Nurs Care Qual*. 2016;31(3):224-232. doi:10.1097/NCQ.0000000000000176.

84. Shin T, Jo I, Choi D, et al. The adverse effect of emergency department crowding on compliance with the resuscitation bundle in the management of severe sepsis and septic shock. *Crit Care*. 2013;17(5):R224. doi:10.1186/cc13047.

85. Sutton D, Windsor J, Husk J. A care bundle approach to falls prevention. *Nurs Times*. 2014;110(20):21-23. http://ovidsp.ovid.com/ovidweb.cgi?T=JS&PAGE=reference&D=medl&NEWS=N&AN=24915673.

86. Takesue Y, Ueda T, Mikamo H, et al. Management bundles for candidaemia: the impact of compliance on clinical outcomes. *J Antimicrob Chemother*. 2015;70(2):587-593. doi:10.1093/jac/dku414.

87. Tanner J, Padley W, Assadian O, Leaper D, Kiernan M, Edmiston C. Do surgical care bundles reduce the risk of surgical site infections in patients undergoing colorectal surgery? A systematic review and cohort meta-analysis of 8,515 patients. *Surgery*. 2015;158(1):66-77. doi:10.1016/j.surg.2015.03.009.

88. Tillman M, Wehbe-Janek H, Hodges B, Smythe WR, Papaconstantinou HT. Surgical care improvement project and surgical site infections: can integration in the surgical safety checklist improve quality performance and clinical outcomes? *J Surg Res*. 2013;184(1):150-156. doi:10.1016/j.jss.2013.03.048.

89. Toltzis P, O’Riordan M, Cunningham DJ, et al. A Statewide Collaborative to Reduce Pediatric Surgical Site Infections. *Pediatrics*. 2014;134(4):e1174-e1180. doi:10.1542/peds.2014-0097.

90. Toth NR, Chambers RM, Davis SL. Implementation of a care bundle for antimicrobial stewardship. *Am J Health Syst Pharm*. 2010;67(9):746-749. doi:10.2146/ajhp090259.

91. Tromp M, Hulscher M, Bleeker-Rovers CP, et al. The role of nurses in the recognition and treatment of patients with sepsis in the emergency department: a prospective before-and-after intervention study. *Int J Nurs Stud*. 2010;47(12):1464-1473. doi:10.1016/j.ijnurstu.2010.04.007.

92. van der Slegt J, van der Laan L, Veen EJ, Hendriks Y, Romme J, Kluytmans J. Implementation of a bundle of care to reduce surgical site infections in patients undergoing vascular surgery. *PLoS One*. 2013;8(8):e71566. doi:10.1371/journal.pone.0071566.

93. Viale P, Scudeller L, Pea F, et al. Implementation of a Meningitis Care Bundle in the Emergency Room Reduces Mortality Associated With Acute Bacterial Meningitis. *Ann Pharmacother*. 2015;49(9):978-985. doi:10.1177/1060028015586012.

94. Wang Z, Xiong Y, Schorr C, Dellinger RP. Impact of sepsis bundle strategy on outcomes of patients suffering from severe sepsis and septic shock in china. *J Emerg Med*. 2013;44(4):735-741. doi:10.1016/j.jemermed.2012.07.084.

95. Westphal GA, Koenig Á, Caldeira Filho M, et al. Reduced mortality after the implementation of a protocol for the early detection of severe sepsis. *J Crit Care*. 2011;26(1):76-81. doi:10.1016/j.jcrc.2010.08.001.

96. Wheeler DS, Giaccone MJ, Hutchinson N, et al. A hospital-wide quality-improvement collaborative to reduce catheter-associated bloodstream infections. *Pediatrics*. 2011;128(4):e995-NaN-7. doi:10.1542/peds.2010-2601.

97. Whippy A, Skeath M, Crawford B, et al. Kaiser Permanente’s performance improvement system, part 3: multisite improvements in care for patients with sepsis. *Jt Comm J Qual Patient Saf*. 2011;37(11):483-493. http://www.ncbi.nlm.nih.gov/pubmed/22132659.

98. Wick EC, Gibbs L, Indorf LA, Varma MG, Garcia-Aguilar J. Implementation of Quality Measures to Reduce Surgical Site Infection in Colorectal Patients. *Dis Colon Rectum*. 2008;51(7):1004-1009. doi:10.1007/s10350-007-9142-y.

99. Wick EC, Hobson DB, Bennett JL, et al. Implementation of a surgical comprehensive unit-based safety program to reduce surgical site infections. *J Am Coll Surg*. 2012;215(2):193-200. doi:10.1016/j.jamcollsurg.2012.03.017.
