# Supplementary material for: Barriers and facilitators to the successful development, implementation and evaluation of care bundles in acute care in hospital: a scoping review
Source: Implement Sci. 2019 May 6;14:47. doi: 10.1186/s13012-019-0894-2 (PMC6501296; doi:10.1186/s13012-019-0894-2)
Supplement: Supplementary file 3 — Appendix 3A. Statistical results table. Appendix 3B. List of studies used for statistical analysis (DOCX 116 kb) [file 13012_2019_894_MOESM3_ESM.docx]

**Supplementary material 3A: Statistics results tables**

| **Element complexity v Compliance** | | |
| --- | --- | --- |
| Compliance V simple/complex elements | Chi-squared=23.051  **p=<0.001** | Kruskal-Wallis rank sum test |

**Table of statistical results: good &fair studies (D&B 14-28)**

| **Implementation strategy v Compliance (n=44 bundles)** | | | | |
| --- | --- | --- | --- | --- |
|  | Unadjusted coefficient | Unadjusted p | Adjusted coefficient * | Adjusted p |
| MDT | 0.0929 | 0.2552 | 0.01903 | 0.8150 |
| Champions | 0.1231 | 0.1293 | 0.14233 | 0.0685 |
| RCA | 0.29106 | **0.03502** | 0.17133 | 0.2160 |
| PDSA cycles | 0.19846 | **0.01695** | 0.13448 | 0.0763 |
| Reminders | -0.16072 | **0.04545** | -0.18326 | **0.0198** |

*Adjusted R^2^ 23% p<0.009

| **Element number v Compliance** | | | |
| --- | --- | --- | --- |
|  | Non-parallel Cohort Study (n=25 bundles) | Prospective Cohort Study (n=12 bundles) | Statistical Test |
| Resultant compliance | Rho= -0.47  **p=0.01672** | Rho= -0.652  **p=0.0241** | Spearman’s rank order correlation |
|  | Non-parallel Cohort Study (n=23 bundles) | Prospective Cohort Study (n=8 bundles) |  |
| Improvement compliance | Rho= -0.619  **p=0.001643** | Rho= 0.16366  p=0.6986 | Spearman’s rank order correlation |

**Table of statistics: poor studies (D&B 0-13)**

| **Implementation strategy v Compliance (n=13)** | | | | |
| --- | --- | --- | --- | --- |
|  | Unadjusted coefficient | Unadjusted p | Adjusted coefficient * | Adjusted p |
| MDT | 0.3488 | **0.0333** | 0.41763 | 0.109 |
| Champions | 0.1183 | 0.533595 | -0.2111 | 0.236 |
| RCA | 0.05252 | 0.767 | 0.15674 | 0.438 |
| PDSA cycles | 0.39257 | **0.01023** | 0.2697 | 0.134 |
| Reminders | -0.37767 | 0.24 | 0.05667 | 0.857 |

*Adjusted R^2^ 45% p=0.093

| **Element number v Compliance** | | | |
| --- | --- | --- | --- |
|  | Non-parallel Cohort Study (n=7 bundles) | Prospective Cohort Study (n=5 bundles) | Statistical Test |
| Resultant compliance | Rho= -0.312  p=0.4957 | Rho= -0.975  **p=0.004818** | Spearman’s rank order correlation |
|  | Non-parallel Cohort Study (n=7) | Prospective Cohort Study (n=1) | Spearman’s rank order correlation |
| Improvement compliance | Rho= -0.03704  p=0.9372 | Rho= N/A  p=N/A |  |

| **Element number v Compliance (n=13)** | | | |
| --- | --- | --- | --- |
|  | Non-parallel Cohort Study (n=7 bundles) | Prospective Cohort Study (n=5 bundles) | Statistical Test |
| Resultant compliance | Rho= -0.312  p=0.4957 | Rho= -0.975  **p=0.004818** | Spearman’s rank order correlation |
|  | Non-parallel Cohort Study (n=7) | Prospective Cohort Study (n=1) | Spearman’s rank order correlation |
| Improvement compliance | Rho= -0.03704  p=0.9372 | Rho= N/A  p=N/A |  |

**Supplementary material 3B: Studies used for statistical analysis**

List of studies used for linear regression of implementation strategy against compliance based on study quality:

Antworth A, Collins CD, Kunapuli A, et al. Impact of an antimicrobial stewardship program comprehensive care bundle on management of candidemia. *Pharmacotherapy*. 2013;33(2):137-143. doi:10.1002/phar.1186.

Bandara S, Lynch G, Cooke C, Varghese P, Ward N. Using Care Bundles to Improve Surgical Outcomes and Reduce Variation in Care for Fragility Hip Fracture Patients. *Geriatr Orthop Surg Rehabil*. 2017;8(2):104-108. doi:10.1177/2151458516681634.

Berg GM, Vasquez DG, Hale LS, Nyberg SM, Moran DA. Evaluation of Process Variations in Noncompliance in the Implementation of Evidence-Based Sepsis Care. *J Healthc Qual*. 2013;35(1):60-69. doi:10.1111/j.1945-1474.2011.00168.x.

Bessesen MT, Lopez K, Guerin K, et al. Comparison of control strategies for methicillin-resistant Staphylococcus aureus. *Am J Infect Control*. 2013;41(11):1048-1052. doi:10.1016/j.ajic.2013.01.032.

Carreno JJ, Kenney RM, Bloome M, et al. Evaluation of pharmacy generalists performing antimicrobial stewardship services. *Am J Heal Pharm*. 2015;72(15):1298-1303. doi:10.2146/ajhp140619.

Casserly B, Baram M, Walsh P, Sucov A, Ward NS, Levy MM. Implementing a collaborative protocol in a sepsis intervention program: Lessons learned. *Lung*. 2011;189(1):11-19. doi:10.1007/s00408-010-9266-z.

Crolla RMPH, van der Laan L, Veen EJ, Hendriks Y, van Schendel C, Kluytmans J. Reduction of surgical site infections after implementation of a bundle of care. *PLoS One*. 2012;7(9):e44599. doi:10.1371/journal.pone.0044599.

Coba V, Whitmill M, Mooney R, et al. Resuscitation bundle compliance in severe sepsis and septic shock: improves survival, is better late than never. *J Intensive Care Med*. 2011;26(5):304-313. doi:10.1177/0885066610392499.

Daniels R, Nutbeam T, McNamara G, Galvin C. The sepsis six and the severe sepsis resuscitation bundle: a prospective observational cohort study. *Emerg Med J*. 2011;28(6):507-512. doi:10.1136/emj.2010.095067.

De Miguel-Yanes JM, Muñoz-González J, Andueza-Lillo J a., Moyano-Villaseca B, González-Ramallo VJ, Bustamante-Fermosel A. Implementation of a bundle of actions to improve adherence to the Surviving Sepsis Campaign guidelines at the ED. *Am J Emerg Med*. 2009;27(6):668-674. doi:10.1016/j.ajem.2008.05.010.

Duffy EA, Rodgers CC, Shever LL, Hockenberry MJ. Implementing a Daily Maintenance Care Bundle to Prevent Central Line-Associated Bloodstream Infections in Pediatric Oncology Patients. *J Pediatr Oncol Nurs*. 2015;(4):1-8. doi:10.1177/1043454214563756.

Ferrer R. Improvement in Process of Care and Outcome After a Multicenter Severe Sepsis Educational Program in Spain. *JAMA*. 2008;299(19):2294. doi:10.1001/jama.299.19.2294.

Girardis M, Rinaldi L, Donno L, et al. Effects on management and outcome of severe sepsis and septic shock patients admitted to the intensive care unit after implementation of a sepsis program: a pilot study. *Crit Care*. 2009;13(5):R143. doi:10.1186/cc8029.

Joslin J, Wilson H, Zubli D, et al. Recognition and management of acute kidney injury in hospitalised patients can be partially improved with the use of a care bundle. *Clin Med (Northfield Il)*. 2015;15(5):431-436. doi:10.7861/clinmedicine.15-5-431.

Kakebeeke D, Vis A, de Deckere ER, Sandel MH, de Groot B. Lack of clinically evident signs of organ failure affects ED treatment of patients with severe sepsis. *Int J Emerg Med*. 2013;6(1):4. doi:10.1186/1865-1380-6-4.

Kang MJ, Shin TG, Jo IJ, et al. Factors influencing compliance with early resuscitation bundle in the management of severe sepsis and septic shock. *Shock*. 2012;38(5):474-479. doi:10.1097/SHK.0b013e31826eea2b.

Kim J, Na S, Yoo YC, Koh SO. Implementing a Sepsis Resuscitation Bundle Improved Clinical Outcome: A Before-and-After Study. *Korean J Crit Care Med*. 2014;29(4):250-256. doi:10.4266/kjccm.2014.29.4.250.

Kleidon T, Illing A, Fogarty G, Edwards R, Tomlinson J, Ullman A. Improving the central venous access devices maintenance process to reduce associated infections in paediatrics: evaluation of a practical, multi-faceted quality-improvement initiative. *Healthc Infect*. 2015;20(2):46. doi:10.1071/HI14038.

Kolhe N V, Staples D, Reilly T, et al. Impact of Compliance with a Care Bundle on Acute Kidney Injury Outcomes: A Prospective Observational Study. *PLoS One*. 2015;10(7):e0132279. doi:10.1371/journal.pone.0132279.

Kolhe N V., Reilly T, Leung J, et al. A simple care bundle for use in acute kidney injury: A propensity score-matched cohort study. *Nephrol Dial Transplant*. 2016;31(11):1846-1854. doi:10.1093/ndt/gfw087.

Kuan WS, Mahadevan M, Tan JH, Guo J, Ibrahim I. Feasibility of introduction and implementation of the Surviving Sepsis Campaign bundle in a Singapore Emergency Department. *Eur J Emerg Med*. 2013;20(5):344-349. doi:10.1097/MEJ.0b013e32835c2ba3.

Laguna-Pérez A, Chilet-Rosell E, Delgado Lacosta M, Alvarez-Dardet C, Uris Selles J, Muñoz-Mendoza CL. Clinical pathway intervention compliance and effectiveness when used in the treatment of patients with severe sepsis and septic shock at an Intensive Care Unit in Spain. *Rev Lat Am Enfermagem*. 2012;20(4):635-643. http://www.ncbi.nlm.nih.gov/pubmed/22990147.

Levy MM, Dellinger RP, Townsend SR, et al. The surviving sepsis campaign: Results of an international guideline-based performance improvement program targeting severe sepsis. *Intensive Care Med*. 2010;36(2):222-231. doi:10.1007/s00134-009-1738-3.

Liu VX, Morehouse JW, Marelich GP, et al. Multicenter implementation of a treatment bundle for patients with sepsis and intermediate lactate values. *Am J Respir Crit Care Med*. 2016;193(11):1264-1270. doi:10.1164/rccm.201507-1489OC.

Nguyen CT, Gandhi T, Chenoweth C, et al. Impact of an antimicrobial stewardship-led intervention for Staphylococcus aureus bacteraemia: a quasi-experimental study. *J Antimicrob Chemother*. 2015;70(12):3390-3396. doi:10.1093/jac/dkv256.

Nguyen HB, Lynch EL, Mou JA, Lyon K, Wittlake WA, Corbett SW. The utility of a quality improvement bundle in bridging the gap between research and standard care in the management of severe sepsis and septic shock in the emergency department. *Acad Emerg Med*. 2007;14(11):1079-1086. doi:10.1197/j.aem.2007.06.024.

Nguyen HB, Kuan W, Batech M, et al. Outcome effectiveness of the severe sepsis resuscitation bundle with addition of lactate clearance as a bundle item: a multi-national evaluation. *Crit Care*. 2011;15(5):R229. doi:10.1186/cc10469.

Noritomi DT, Ranzani OT, Monteiro MB, et al. Implementation of a multifaceted sepsis education program in an emerging country setting: clinical outcomes and cost-effectiveness in a long-term follow-up study. *Intensive Care Med*. 2014;40(2):182-191. doi:10.1007/s00134-013-3131-5.

Pastor C, Artinyan A, Varma MG, Kim E, Gibbs L, Garcia-Aguilar J. An Increase in Compliance With the Surgical Care Improvement Project Measures Does Not Prevent Surgical Site Infection in Colorectal Surgery. *Dis Colon Rectum*. 2010;53(1):24-30. doi:10.1007/DCR.0b013e3181ba782a.

Plambech MZ, Lurie AI, Ipsen HL. Initial, successful implementation of sepsis guidelines in an emergency department. *Dan Med J*. 2012;59(12).

Power M, Tyrrell PJ, Rudd AG, et al. Did a quality improvement collaborative make stroke care better? A cluster randomized trial. *Implement Sci*. 2014;9(1):40. doi:10.1186/1748-5908-9-40.

Ramsdell TH, Smith AN, Kerkhove E. Compliance with Updated Sepsis Bundles to Meet New Sepsis Core Measure in a Tertiary Care Hospital. *Hosp Pharm*. 2017;52(3):177-186. doi:10.1310/hpj5203-177.

Rinke ML, Chen AR, Bundy DG, et al. Implementation of a Central Line Maintenance Care Bundle in Hospitalized Pediatric Oncology Patients. *Pediatrics*. 2012;130(4):e996-e1004. doi:10.1542/peds.2012-0295.

Secola R, Lewis MA, Pike N, Needleman J, Doering L. Feasibility of the use of a reliable and valid central venous catheter blood draw bundle checklist. *J Nurs Care Qual*. 2012;27(3):218-225. doi:10.1097/NCQ.0b013e3182461fab.

Seoane L, Winterbottom F, Nash T, et al. Using quality improvement principles to improve the care of patients with severe sepsis and septic shock. *Ochsner J*. 2013;13(3):359-366. http://www.ncbi.nlm.nih.gov/pubmed/24052765.

Shin T, Jo I, Choi D, et al. The adverse effect of emergency department crowding on compliance with the resuscitation bundle in the management of severe sepsis and septic shock. *Crit Care*. 2013;17(5):R224. doi:10.1186/cc13047.

Takesue Y, Ueda T, Mikamo H, et al. Management bundles for candidaemia: the impact of compliance on clinical outcomes. *J Antimicrob Chemother*. 2015;70(2):587-593. doi:10.1093/jac/dku414.

Tanner J, Padley W, Assadian O, Leaper D, Kiernan M, Edmiston C. Do surgical care bundles reduce the risk of surgical site infections in patients undergoing colorectal surgery? A systematic review and cohort meta-analysis of 8,515 patients. *Surgery*. 2015;158(1):66-77. doi:10.1016/j.surg.2015.03.009.

Toth NR, Chambers RM, Davis SL. Implementation of a care bundle for antimicrobial stewardship. *Am J Health Syst Pharm*. 2010;67(9):746-749. doi:10.2146/ajhp090259.

Tromp M, Hulscher M, Bleeker-Rovers CP, et al. The role of nurses in the recognition and treatment of patients with sepsis in the emergency department: a prospective before-and-after intervention study. *Int J Nurs Stud*. 2010;47(12):1464-1473. doi:10.1016/j.ijnurstu.2010.04.007.

van der Slegt J, van der Laan L, Veen EJ, Hendriks Y, Romme J, Kluytmans J. Implementation of a bundle of care to reduce surgical site infections in patients undergoing vascular surgery. *PLoS One*. 2013;8(8):e71566. doi:10.1371/journal.pone.0071566.

Wang Z, Xiong Y, Schorr C, Dellinger RP. Impact of sepsis bundle strategy on outcomes of patients suffering from severe sepsis and septic shock in china. *J Emerg Med*. 2013;44(4):735-741. doi:10.1016/j.jemermed.2012.07.084.

Westphal GA, Koenig Á, Caldeira Filho M, et al. Reduced mortality after the implementation of a protocol for the early detection of severe sepsis. *J Crit Care*. 2011;26(1):76-81. doi:10.1016/j.jcrc.2010.08.001.

List of studies not used for linear regression of implementation strategy against compliance due to poor quality:

Baldwin LN, Smith SA, Fender V, Gisby S, Fraser J. An audit of compliance with the sepsis resuscitation care bundle in patients admitted to A&amp;E with severe sepsis or septic shock. *Int Emerg Nurs*. 2008;16(4):250-256. doi:10.1016/j.ienj.2008.05.008.

Bull A, Wilson J, Worth LJ, et al. A bundle of care to reduce colorectal surgical infections: An Australian experience. *J Hosp Infect*. 2011;78(4):297-301. doi:10.1016/j.jhin.2011.03.029.

Bundy DG, Gaur AH, Billett AL, He B, Colantuoni EA, Miller MR. Preventing CLABSIs among pediatric hematology/oncology inpatients: national collaborative results. *Pediatrics*. 2014;134(6):e1678-85. doi:10.1542/peds.2014-0582.

Choi SW, Chang L, Hanauer DA, et al. Rapid reduction of central line infections in hospitalized pediatric oncology patients through simple quality improvement methods. *Pediatr Blood Cancer*. 2013;60(2):262-269. doi:10.1002/pbc.24187.

Fisher JC, Godfried DH, Lighter-Fisher J, et al. A novel approach to leveraging electronic health record data to enhance pediatric surgical quality improvement bundle process compliance. *J Pediatr Surg*. 2016;51(6):1030-1033. doi:10.1016/j.jpedsurg.2016.02.080.

Hettige R, Arora A, Ifeacho S, Narula A. Improving tracheostomy management through design, implementation and prospective audit of a care bundle: how we do it. *Clin Otolaryngol*. 2008;33(5):488-491. doi:10.1111/j.1749-4486.2008.01725.x.

Richardson DA, Bhagwat A, Forster K, et al. The Royal College of Physicians’ Fallsafe care bundles applied trustwide: The Northumbria experience 2013. *Clin Med J R Coll Physicians London*. 2015;15(6):530-535. doi:10.7861/clinmedicine.15-6-530.

Wheeler DS, Giaccone MJ, Hutchinson N, et al. A hospital-wide quality-improvement collaborative to reduce catheter-associated bloodstream infections. *Pediatrics*. 2011;128(4):e995-NaN-7. doi:10.1542/peds.2010-2601.

Whippy A, Skeath M, Crawford B, et al. Kaiser Permanente’s performance improvement system, part 3: multisite improvements in care for patients with sepsis. *Jt Comm J Qual Patient Saf*. 2011;37(11):483-493. http://www.ncbi.nlm.nih.gov/pubmed/22132659.

List of studies used for Spearman’s test (element number versus compliance)

Cohort studies:

Bessesen MT, Lopez K, Guerin K, et al. Comparison of control strategies for methicillin-resistant Staphylococcus aureus. *Am J Infect Control*. 2013;41(11):1048-1052. doi:10.1016/j.ajic.2013.01.032.

Casserly B, Baram M, Walsh P, Sucov A, Ward NS, Levy MM. Implementing a collaborative protocol in a sepsis intervention program: Lessons learned. *Lung*. 2011;189(1):11-19. doi:10.1007/s00408-010-9266-z.

Coba V, Whitmill M, Mooney R, et al. Resuscitation bundle compliance in severe sepsis and septic shock: improves survival, is better late than never. *J Intensive Care Med*. 2011;26(5):304-313. doi:10.1177/0885066610392499.

Daniels R, Nutbeam T, McNamara G, Galvin C. The sepsis six and the severe sepsis resuscitation bundle: a prospective observational cohort study. *Emerg Med J*. 2011;28(6):507-512. doi:10.1136/emj.2010.095067.

Girardis M, Rinaldi L, Donno L, et al. Effects on management and outcome of severe sepsis and septic shock patients admitted to the intensive care unit after implementation of a sepsis program: a pilot study. *Crit Care*. 2009;13(5):R143. doi:10.1186/cc8029.

Kakebeeke D, Vis A, de Deckere ER, Sandel MH, de Groot B. Lack of clinically evident signs of organ failure affects ED treatment of patients with severe sepsis. *Int J Emerg Med*. 2013;6(1):4. doi:10.1186/1865-1380-6-4.

Kuan WS, Mahadevan M, Tan JH, Guo J, Ibrahim I. Feasibility of introduction and implementation of the Surviving Sepsis Campaign bundle in a Singapore Emergency Department. *Eur J Emerg Med*. 2013;20(5):344-349. doi:10.1097/MEJ.0b013e32835c2ba3.

Levy MM, Dellinger RP, Townsend SR, et al. The surviving sepsis campaign: Results of an international guideline-based performance improvement program targeting severe sepsis. *Intensive Care Med*. 2010;36(2):222-231. doi:10.1007/s00134-009-1738-3.

Pastor C, Artinyan A, Varma MG, Kim E, Gibbs L, Garcia-Aguilar J. An Increase in Compliance With the Surgical Care Improvement Project Measures Does Not Prevent Surgical Site Infection in Colorectal Surgery. *Dis Colon Rectum*. 2010;53(1):24-30. doi:10.1007/DCR.0b013e3181ba782a.

Seoane L, Winterbottom F, Nash T, et al. Using quality improvement principles to improve the care of patients with severe sepsis and septic shock. *Ochsner J*. 2013;13(3):359-366. http://www.ncbi.nlm.nih.gov/pubmed/24052765.

Takesue Y, Ueda T, Mikamo H, et al. Management bundles for candidaemia: the impact of compliance on clinical outcomes. *J Antimicrob Chemother*. 2015;70(2):587-593. doi:10.1093/jac/dku414.

Non-parellel cohort studies:

Antworth A, Collins CD, Kunapuli A, et al. Impact of an antimicrobial stewardship program comprehensive care bundle on management of candidemia. *Pharmacotherapy*. 2013;33(2):137-143. doi:10.1002/phar.1186.

Bandara S, Lynch G, Cooke C, Varghese P, Ward N. Using Care Bundles to Improve Surgical Outcomes and Reduce Variation in Care for Fragility Hip Fracture Patients. *Geriatr Orthop Surg Rehabil*. 2017;8(2):104-108. doi:10.1177/2151458516681634.

Carreno JJ, Kenney RM, Bloome M, et al. Evaluation of pharmacy generalists performing antimicrobial stewardship services. *Am J Heal Pharm*. 2015;72(15):1298-1303. doi:10.2146/ajhp140619.

Crolla RMPH, van der Laan L, Veen EJ, Hendriks Y, van Schendel C, Kluytmans J. Reduction of surgical site infections after implementation of a bundle of care. *PLoS One*. 2012;7(9):e44599. doi:10.1371/journal.pone.0044599.

De Miguel-Yanes JM, Muñoz-González J, Andueza-Lillo J a., Moyano-Villaseca B, González-Ramallo VJ, Bustamante-Fermosel A. Implementation of a bundle of actions to improve adherence to the Surviving Sepsis Campaign guidelines at the ED. *Am J Emerg Med*. 2009;27(6):668-674. doi:10.1016/j.ajem.2008.05.010.

Duffy EA, Rodgers CC, Shever LL, Hockenberry MJ. Implementing a Daily Maintenance Care Bundle to Prevent Central Line-Associated Bloodstream Infections in Pediatric Oncology Patients. *J Pediatr Oncol Nurs*. 2015;(4):1-8. doi:10.1177/1043454214563756.

Ferrer R. Improvement in Process of Care and Outcome After a Multicenter Severe Sepsis Educational Program in Spain. *JAMA*. 2008;299(19):2294. doi:10.1001/jama.299.19.2294.

Joslin J, Wilson H, Zubli D, et al. Recognition and management of acute kidney injury in hospitalised patients can be partially improved with the use of a care bundle. *Clin Med (Northfield Il)*. 2015;15(5):431-436. doi:10.7861/clinmedicine.15-5-431.

Kleidon T, Illing A, Fogarty G, Edwards R, Tomlinson J, Ullman A. Improving the central venous access devices maintenance process to reduce associated infections in paediatrics: evaluation of a practical, multi-faceted quality-improvement initiative. *Healthc Infect*. 2015;20(2):46. doi:10.1071/HI14038.

Kolhe N V, Staples D, Reilly T, et al. Impact of Compliance with a Care Bundle on Acute Kidney Injury Outcomes: A Prospective Observational Study. *PLoS One*. 2015;10(7):e0132279. doi:10.1371/journal.pone.0132279.

Laguna-Pérez A, Chilet-Rosell E, Delgado Lacosta M, Alvarez-Dardet C, Uris Selles J, Muñoz-Mendoza CL. Clinical pathway intervention compliance and effectiveness when used in the treatment of patients with severe sepsis and septic shock at an Intensive Care Unit in Spain. *Rev Lat Am Enfermagem*. 2012;20(4):635-643. <http://www.ncbi.nlm.nih.gov/pubmed/22990147>.

Liu VX, Morehouse JW, Marelich GP, et al. Multicenter implementation of a treatment bundle for patients with sepsis and intermediate lactate values. *Am J Respir Crit Care Med*. 2016;193(11):1264-1270. doi:10.1164/rccm.201507-1489OC.

Nguyen CT, Gandhi T, Chenoweth C, et al. Impact of an antimicrobial stewardship-led intervention for Staphylococcus aureus bacteraemia: a quasi-experimental study. *J Antimicrob Chemother*. 2015;70(12):3390-3396. doi:10.1093/jac/dkv256.

Nguyen HB, Lynch EL, Mou JA, Lyon K, Wittlake WA, Corbett SW. The utility of a quality improvement bundle in bridging the gap between research and standard care in the management of severe sepsis and septic shock in the emergency department. *Acad Emerg Med*. 2007;14(11):1079-1086. doi:10.1197/j.aem.2007.06.024.

Noritomi DT, Ranzani OT, Monteiro MB, et al. Implementation of a multifaceted sepsis education program in an emerging country setting: clinical outcomes and cost-effectiveness in a long-term follow-up study. *Intensive Care Med*. 2014;40(2):182-191. doi:10.1007/s00134-013-3131-5.

Plambech MZ, Lurie AI, Ipsen HL. Initial, successful implementation of sepsis guidelines in an emergency department. *Dan Med J*. 2012;59(12).

Ramsdell TH, Smith AN, Kerkhove E. Compliance with Updated Sepsis Bundles to Meet New Sepsis Core Measure in a Tertiary Care Hospital. *Hosp Pharm*. 2017;52(3):177-186. doi:10.1310/hpj5203-177.

Rinke ML, Chen AR, Bundy DG, et al. Implementation of a Central Line Maintenance Care Bundle in Hospitalized Pediatric Oncology Patients. *Pediatrics*. 2012;130(4):e996-e1004. doi:10.1542/peds.2012-0295.

Tanner J, Padley W, Assadian O, Leaper D, Kiernan M, Edmiston C. Do surgical care bundles reduce the risk of surgical site infections in patients undergoing colorectal surgery? A systematic review and cohort meta-analysis of 8,515 patients. *Surgery*. 2015;158(1):66-77. doi:10.1016/j.surg.2015.03.009.

Toth NR, Chambers RM, Davis SL. Implementation of a care bundle for antimicrobial stewardship. *Am J Health Syst Pharm*. 2010;67(9):746-749. doi:10.2146/ajhp090259.

Tromp M, Hulscher M, Bleeker-Rovers CP, et al. The role of nurses in the recognition and treatment of patients with sepsis in the emergency department: a prospective before-and-after intervention study. *Int J Nurs Stud*. 2010;47(12):1464-1473. doi:10.1016/j.ijnurstu.2010.04.007.

van der Slegt J, van der Laan L, Veen EJ, Hendriks Y, Romme J, Kluytmans J. Implementation of a bundle of care to reduce surgical site infections in patients undergoing vascular surgery. *PLoS One*. 2013;8(8):e71566. doi:10.1371/journal.pone.0071566.

Wang Z, Xiong Y, Schorr C, Dellinger RP. Impact of sepsis bundle strategy on outcomes of patients suffering from severe sepsis and septic shock in china. *J Emerg Med*. 2013;44(4):735-741. doi:10.1016/j.jemermed.2012.07.084.

Westphal GA, Koenig Á, Caldeira Filho M, et al. Reduced mortality after the implementation of a protocol for the early detection of severe sepsis. *J Crit Care*. 2011;26(1):76-81. doi:10.1016/j.jcrc.2010.08.001.
